# Hormone-Like Effects of 4-Vinylcyclohexene Diepoxide on Follicular Development

**DOI:** 10.3389/fcell.2020.00587

**Published:** 2020-07-31

**Authors:** Lian Bao Cao, Hong Bin Liu, Gang Lu, Yue Lv, Chi Kwan Leung, Yan Zhi Du, Wu Ming Wang, Zhi Qiang Xiong, Xian Wei Su, Hong Jian Li, Zi-Jiang Chen, Jin Long Ma, Wai Yee Chan

**Affiliations:** ^1^Center for Reproductive Medicine, Cheeloo College of Medicine, Shandong University, Jinan, China; ^2^CUHK-SDU Joint Laboratory on Reproductive Genetics, School of Biomedical Sciences, The Chinese University of Hong Kong, Hong Kong, China; ^3^National Research Center for Assisted Reproductive Technology and Reproductive Genetics, Jinan, China; ^4^SDIVF R&D Centre, Hong Kong Science and Technology Parks, Sha Tin, China; ^5^Center for Reproductive Medicine, Renji Hospital, School of Medicine, Shanghai Jiao Tong University, Shanghai, China

**Keywords:** VCD, ovotoxicity, folliculogenesis, ovulation induction, PI3K-Akt pathway

## Abstract

**Background:**

4-vinylcyclohexene diepoxide (VCD) has long been considered a hazardous occupational chemical that promotes ovarian failure. However, VCD is also used as a research compound to chemically induce animal models of premature ovarian insufficiency (POI), and in related work we unexpectedly found that VCD apparently exhibits both dose- and duration-dependent opposing, hormone-like effects on the maintenance of the primordial follicle pool, follicle development, and ovulation induction.

**Results:**

We conducted experiments with cultured murine ovaries and performed transplantation experiments using postnatal day (PD) 2 and PD12 mice and found that low-dose, short-term exposure to VCD (VCD^low^) actually protects the primordial/primary follicle pool and improves the functional ovarian reserve (FOR) by disrupting follicular atresia. VCD^low^ inhibits follicular apoptosis and regulates the Pten-PI3K-Foxo3a pathway. Short-term VCD exposure *in vivo* (80 mg/kg, 5 days) significantly increases the number of superovulated metaphase II oocytes, preovulatory follicles, and corpus luteum in middle-aged mice with diminished ovarian reserve (DOR). We demonstrate that low-dose but not high-dose VCD promotes aromatase levels in granulosa cells (GCs), thereby enhancing the levels of estradiol secretion.

**Conclusion:**

Our study illustrates a previously unappreciated, hormone-like action for the occupational “ovotoxin” molecule VCD and strongly suggests that VCD^low^ should be explored for its potential utility for treating human ovarian follicular development disorders, including subfertility in perimenopausal women.

## Introduction

An ovary contains a finite pool of oocyte-containing follicles at different developmental stages, and this pool of dormant primordial follicles provides developing follicles and oocytes, known as the functional ovarian reserve (FOR) over the entire female reproductive lifespan as controlled by numerous regulatory signals (Adhikari and Liu, 2009). Successive follicular waves during interovulatory intervals function to coordinate folliculogenesis, ultimately facilitating the recruitment and preferential growth of one dominant follicle leading up to ovulation (de Mello Bianchi et al., 2010). The FOR diminishes with age and results in the termination of the reproductive lifespan at approximately 50 years of age. Women with diminished ovarian reserve (DOR) may experience menopausal symptoms including failure of follicle maturation and a significant decrease in ovarian gonad steroids such as estrogen, leading to a multitude of complications, e.g., cardiovascular diseases, osteoporosis, and cognitive impairment (Buckler, 2005). Premature ovarian insufficiency (POI) patients may develop more severe menopausal symptoms compared to older menopausal women (Torrealday et al., 2017).

A growing list of natural and anthropogenic xenobiotics has been shown to perturb ovarian metabolism, menstrual cycles, and follicular development (Bhattacharya and Keating, 2012). These ovotoxicants have been shown to deregulate endocrine signaling, redox homeostasis, and epigenetic modifications and to impair ovarian functions and trigger broad or targeted follicular atresia, and ultimately induce DOR (Vabre et al., 2017).

4-vinylcyclohexene diepoxide (VCD) is an ovotoxic catabolite of the occupational chemical 4-vinylcyclohexene, an intermediate used in the manufacture of flame retardants, flavors, pesticides, adhesives, fragrances, and synthetic rubber (Rappaport and Fraser, 1977; Devine et al., 2001). Prior studies have consistently indicated that VCD induces atretic degeneration of primordial and primary follicles in rodents and primates (Devine et al., 2002; Naz, 2005; Appt et al., 2006). Such VCD-induced ovotoxicity has been associated with the B-cell lymphoma 2 (Bcl-2) pro-apoptotic signaling pathway, with kit ligand signaling, with the rapamycin-insensitive companion of mammalian target of rapamycin (rictor)/mTORC2 pathway, and with the nuclear factor erythroid 2-related factor 2-mediated oxidative stress response pathway (Springer et al., 1996; Hu et al., 2001, 2006; Fernandez et al., 2008).

As a research compound, VCD is commonly used to chemically induce animal models of POI, one of the common causes of female infertility. VCD depletes the FOR, enabling the study of the etiopathogenesis of this reproductive disorder (Chen et al., 2015). However, it is notable that although a standard treatment regime of daily VCD (80 or 160 mg/kg for 10–15 days) in postnatal day (PD) 28 animals via intraperitoneal injection (IP) is widely adopted to elicit follicular atresia (Kappeler and Hoyer, 2012), such concentrations (equivalent to roughly 1 mg/ml in blood) are several orders of magnitude higher than the reported serum/blood concentrations of several well-known endocrine-disrupting chemicals or ovotoxicants in human occupational exposure studies. The low dose is considered to have no effect either *in vivo* or *in vitro*. All of these attributes are inconsistent with the definition of VCD as an environmental pollutant in which the body may react at a much lower dosage such as bisphenol A. In the clinic, VCD exposure has long been considered a likely culprit for POI in occupationally exposed individuals, although there is no direct clinical data to support this supposition (Hu et al., 2006).

Here, we aimed to systematically examine the dose- and duration-dependent hormone-like effects on follicle development, maturation, and ovulation induction. We examined *in vitro* ovary cultures and performed ovary transplantation experiments to investigate the effects of different VCD doses on follicular development *in vitro* and *in vivo* in C57BL/6J female mice at PD2 and PD12 and in adult (2 months old) and middle-aged (10–12 months old) mice with DOR. We found that very short-term (2–3 min) VCD exposure (VCD^low^) can activate key proteins in the PI3K-Akt-Foxo3a and mTOR pathways during ovarian culture. However, the expression of several transcription factors including *Foxo3a* and *Foxl2*, which are positively regulated by short-term VCD, began to increase within 6 h of treatment. Simultaneously, the expression of numerous transcription factors (*Figla*, *Nobox*, *Gdf9*, *LHX8*, *Sohlh1*, and *Sohlh2*) known to positively regulate early follicular development were downregulated by 6 h. Biochemically, we demonstrate that short-term VCD can stimulate granulosa cells (GCs) to express aromatase, which catalyzes estrogen production and promotes follicular development and maturation (Fisher et al., 1998). We also evaluated the effects of short-term VCD exposure *in vivo* (80 mg/kg VCD for 5 days) on the concentration of serum FSH and E2, as well as the number of superovulated metaphase II (MII) oocytes, preovulatory follicles, and corpus luteum in middle-aged DOR mice. Our work supports that VCD confers hormone-like endocrine effects, suggesting the utility of exploring VCD as an alternative therapeutic strategy for POI patients. Fundamentally, short-term VCD exposure may be a tool for improving subfertility in perimenopausal women.

## Materials and Methods

### Chemical

4-vinylcyclohexene diepoxide was purchased from Sigma Aldrich (Cat# 94956-100 ML). For cell culture, *in vitro* maturation, and *in vitro* fertilization, VCD was dissolved in phosphate-buffered saline (PBS) (Cat# 10010-0323, Gibco). For intraperitoneal injection, VCD was dissolved in normal saline (0.9% sodium chloride). *bpV* (HOpic) was purchased from Selleckchem (Cat# S8651-5 mg).

### Animals

**FIGURE 1:**
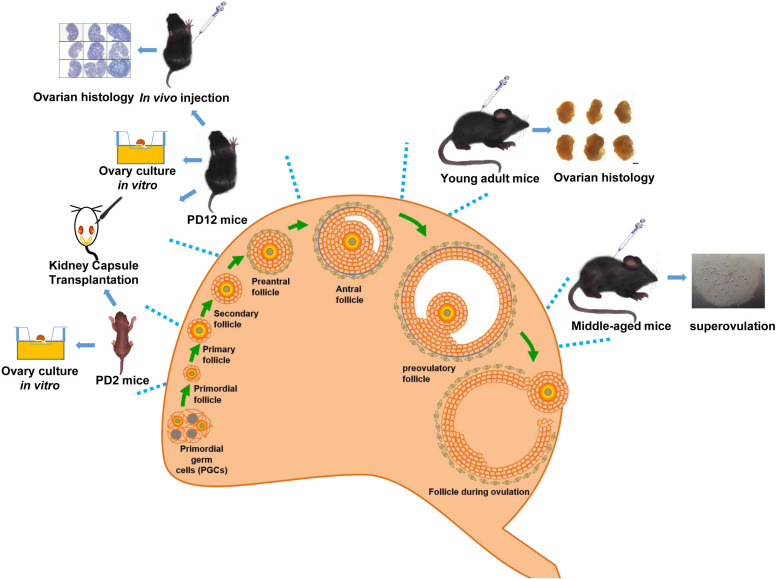
Schematic diagram of the overall experimental design.

Wild-type C57BL/6J female mice (PD2, PD12, PD28, 2 months, and 10–12 months of age) were sourced from the Laboratory Animal Experimental Service Center at the Chinese University of Hong Kong (CUHK) and the Laboratory Animal Center of Shandong University. The Figure 1 shows the age of mice selected at different stages of follicular development. Animal handlings and experimental procedures in Hong Kong were performed in accordance with CUHK Schedule 7 Regulations on Use of Experimental Animals, the International Guiding Principles for Biomedical Research Involving Animals, the Hong Kong Code of Practice for Care and Use of Animals for Experimental Purposes, and the CUHK Guide for Animal Care Use. Animal handlings and experimental procedures in Shandong were conducted in conformity to the “National Institute of Health Guide for the Care and Use of Laboratory Animals revised 1996.”

The animals were housed in an air-conditioned (22 ± 1°C) environment with 45–55% humidity and a 12-h alternating light-dark cycle. Standard lab feed and water were given *ad libitum*. All animal experimental procedures were approved by the University Animal Experiments Ethics Committee of the CUHK. For the animal experiments in Shandong University, all procedures were approved by the Ethics Review Board of the Center for Reproductive Medicine of Shandong University.

### Ovary Culture *in vitro*

Paired ovaries from euthanized PD2 or PD12 C57BL/6J female mice were dissected and washed three times in Leibovitz’s L-15 medium (Cat# 41400-045, Gibco) containing 10% fetal bovine serum (FBS) before being transferred individually to cell culture inserts (Cat# PICMORG50, Millipore) in a 6-well culture plate (Cat# 3506, Costar). One ovary was incubated with VCD and the contralateral ovary served as the paired control. Each cell culture insert housed three dissected ovaries. Ovaries were cultured in 1.5 ml Dulbecco’s modified Eagle’s medium/Ham’s F12 (DMEM/F12) medium (Cat# 11039-021, Gibco) supplemented with 1 mg/ml BSA (Cat# A4161-1G, Sigma), 1 mg/ml AlbuMAX II Lipid-Rich BSA (Cat# 11021029, Gibco), 5% Insulin-Transferrin-Selenium (ITS-G) (Cat# 41400-045, Gibco), and 1% penicillin-streptomycin at 37°C with 5% CO_2_ concentration and ∼95% relative humidity under normal atmosphere. The paraffin-embedded ovaries were serially sectioned into 5 μm thickness using a microtome and stained with hematoxylin for histological analysis.

### Ovary Transplantation Under the Kidney Capsule

The kidneys of the anesthetized host animals were externalized through a dorso- longitudinal incision. The paired ovaries (VCD-treated and paired controls) from the same donor were excised and separately allografted into two 2-month old host mice followed by IP injection of VCD at 80 mg/kg for three consecutive days. The age-matched paired control groups were treated with an equal amount of 0.9% normal saline. Starting at 3 days after ovary transplantation donated by PD12 mice, the hosts were administrated 2 IU of follicle-stimulating hormone (FSH) per mouse via daily IP injection, and the grafts were harvested 7 or 14 days later for histological examination. No FSH treatment was given to the host mice that received ovarian grafts from PD2 donors.

### Follicle Counting and Identification

Ovarian grafts were fixed in 4% buffered paraformaldehyde (Cat# P6148, Sigma) overnight, embedded in paraffin, serially sectioned into 5 μm thickness using a microtome, and hematoxylin stained. Only follicles with a stained nucleus in the oocyte were counted.

Follicles were categorized according to their maturation stage – a primordial follicle contained an oocyte surrounded by a single layer of squamous GCs; a primary follicle was characterized by a layer of cuboidal GCs around the oocyte (a layer of cuboidal GCs accompanied by oocyte enlargement was considered a late primary follicle); a secondary follicle was surrounded by more than two layers of cuboidal GCs, with an enlarged oocyte; a “preantral follicle” (early antral follicle) was a large secondary follicle with multiple layers of GCs with a developing antrum; and mature preovulatory follicles had a large central antrum.

### RNA Extraction and qPCR

The entire ovarian tissue was digested in QIAzol Lysis Reagent (Cat# 79306, QIAGEN) for 20 min, and the total mRNA was extracted using an RNA extraction kit (Cat# R2052, Zymo Research). The reverse transcription reaction was performed using GoScript^TM^ Reverse Transcriptase (Cat# A2790, Promega).

For single-oocyte qPCR, the ovaries from PD12 mice were digested and the follicles were isolated according to size as primordial follicles (20 μm), primary follicles (40–50 μm), and secondary follicles (70 μm). For single-oocyte qPCR, follicles at various maturation stages were collected and lysed using a Single Cell Lysis Kit according to the manufacturer’s protocol (Cat# PN 4458235, Thermo Scientific). The reverse transcription reaction was performed using a SuperScript^®^ VILO^TM^ cDNA Synthesis Kit with a 10-μl volume (Cat# 11754-050, Thermo Scientific). The RNA concentration was measured using a NanoDrop spectrophotometer (Thermo Scientific, United States).

Quantitative polymerase chain reaction (qPCR) was performed using the QuantStudio 7 Flex Real-time PCR System (Applied Biosystems) per the manufacturer’s instructions. The real-time PCR process used SYBR Green Master Premix Ex Taq (Takara, Japan). The thermocycling conditions were as follows: denaturation at 95°C for 30 s, 40 cycles of denaturation at 95°C for 5 s, and annealing/extension at 60°C for 34 s. Relative gene expression between the VCD-treated and paired control samples was calculated using the 2^–ΔΔCT^ method with *glyceraldehyde 3-phosphate dehydrogenase* (*Gapdh*) as an endogenous control. The primer sequences are shown in Table 1.

**TABLE 1:** List of primer sequences used for qPCR analysis in this study.

**Gene name**	**Forward primer sequence**	**Reverse primer sequence**	**Size**
	**(5′ to 3′)**	**(5′ to 3′)**	**(bp)**
*Bcl-2*	GAACTGGGGGAGGATTGTGG	GCATGCTGGGGCCATATAGT	194
*Bax*	TGAAGACAGGGGCCTTTTTG	AATTCGCCGGAGACACTCG	140
*Gapdh*	GAGAGTGTTTCCTCGTCCCG	ACTGTGCCGTTGAATTTGCC	201
*Figla*	TGACCACCATGGATACAGCA	TGTGGTAGAAACGGCACCAG	280
*Foxo3*	CTGGGGGAACCTGTCCTATG	TCATTCTGAACGCGCATGAAG	210
*Gdf9*	TCTTAGTAGCCTTAGCTCTCAGG	TGTCAGTCCCATCTACAGGCA	116
*Bmp15*	TCCTTGCTGACGACCCTACAT	TACCTCAGGGGATAGCCTTGG	100
*Nobox*	ATGGAACCTACGGAGAAGCTC	CTCAGAGGTCTTCGACAGTGG	189
*Sohlh1*	CGGGCCAATGAGGATTACAGA	TCCTGCGTTCTCTCTCGCT	181
*Sohlh2*	GGGCAGGGCAGAGTAAATCTT	CAAACGAGTTAGCAGCCAAAAG	154
*Foxl2*	ACAACACCGGAGAAACCAGAC	*CGTAGAACGGGAACTTGGCTA*	145
*Lhx8*	AGGCAAGTGTGTGTGCAGCA	GATTGTCCAGCATGCAGTCA	380

### Ovulation Stimulation

Aged female mice received one IP injection of 5 IU PMSG. Forty-eight hours later, another injection of 10 IU hCG was administered. Sixteen hours later, MII oocytes were collected from the oviducts and placed into a hyaluronidase solution (Cat# 90101, Fujifilm Irvine Scientific) to remove the cumulus cells surrounding the retrieved oocytes. The mice in the experimental group were IP injected with VCD every morning and given 5 IU of PMSG in the afternoon on the fifth treatment day. Saline was given to the drug-paired control mice.

### Hormone Measurement

The FSH and estradiol (E2) levels in serum of 10–12-month-old mice were determined using an XH6080 Radioimmunoassay system (Xi’an Nuclear Instrument Factory, China). The E2 level in the supernatant from the human ovarian granulosa-like tumor KGN cell culture and ovarian culture were determined using a Human Estradiol ELISA Kit (Cat# KAQ0621, Invitrogen).

### Immunoblotting

The ovarian samples were homogenized in RIPA Lysis Buffer (Cat# 89900, Thermo) with protease inhibitors (Cat# B14002, Bimake), then centrifuged at 14,000 × *g* for 15 min at 4°C. The supernatant was collected, and the protein concentrations were measured using a Pierce BCA Protein Assay Kit (Cat# 23225, Thermo). The protein sample was heated to 100°C for 10 min for denaturation. Equal amounts of proteins (10–20 μg) were separated via 10% sodium dodecyl sulfate-polyacrylamide gel electrophoresis (SDS-PAGE) (Cat# 456-1069, Bio-Rad) and transferred to Protran nitrocellulose membranes (Cat# IPVH00010, Immobilon-P). The membranes were blocked for 1 h with 5% non-fat dry milk in PBST (10% PBS with 0.1% Tween 20, pH 7.6), followed by incubation with the following primary antibodies overnight at 4°C: anti-rabbit aromatase (Cat# NBP1-45360, Novus, 1:2000), anti-rabbit PTEN (D4.3) (Cat# 9188, Cell Signaling, 1:1000), anti-rabbit phosphor-AKT (Serine 473) (D9E) (Cat# 4060, Cell Signaling, 1:500), anti-rabbit AKT (pan) (C67E7) (Cat# 4691, Cell Signaling, 1:1000), anti-rabbit FoxO3a (75D8) (Cat# 2497, Cell Signaling, 1:1000), anti-rabbit phosphor-FoxO3a (Serine 253) (Cat# 9466, Cell Signaling, 1:500), anti-rabbit phosphor-FoxO1 (Threonine 24)/ FoxO3a (Threonine 32) (Cat# 9464, Cell Signaling, 1:500), anti-rabbit phosphor-P-S6 (Serine 235/236) (Cat# 4858, Cell Signaling, 1:1000), anti-rabbit phosphor-P-S6 (Serine 240/244) (Cat# 2215, Cell Signaling, 1:1000), anti-rabbit P-S6 (Cat# 2217, Cell Signaling, 1:1000), anti-rabbit phosphor-P70S6k (Threonine 389) (Cat# 9205, Cell Signaling, 1:1000), anti-mouse Bcl-2 (C-2) (Cat# Sc-7382, Santa Cruz, 1:1000), and anti-Bax (Cat# 2772, Cell Signaling, 1:1000). The membranes were washed three times with PBST for 10 min and were incubated with the anti-rabbit (Cat# 7074S, CST) or anti-mouse (Cat# 7076S, CST) Immunoglobulin G (IgG) (heavy and light chain) antibody conjugated to horseradish peroxidase (HRP) secondary antibody at a dilution of 1:5,000 in PBST for 1 h at room temperature. The membranes were washed three times with PBST for 10 min. The blot was then developed using an Enhanced Chemiluminescence Plus detection kit (Cat. #: K-12045-D20, Advansta, United States) and visualized using a Kodak Medical X-ray Processor 102 (Cat# K-07014-100, Kodak). The protein bands were quantified using ImageJ software and expressed as the densitometric quantization of the protein band intensities normalized against the corresponding β-actin band intensities. This experiment was repeated in triplicate, and representative blot images are presented.

### Immunohistochemistry Staining

The immunostaining was performed as previously described (Li et al., 2010). Briefly, VCD-treated ovaries were fixed in 10% buffered formalin for 12 h, and 5 μm slices of paraffin-embedded ovarian tissues were deparaffinized and rehydrated and subjected to antigen retrieval pretreatment. The tissues were immunostained with the primary antibodies overnight at 4°C: anti-aromatase antibody (Cat# ab18995, Abcam, 1:400), anti-FOXO3a antibody (Cat# 2497, CST, 1:400), and anti-cleaved CASPASE-3 (Cat# 9664, CST, 1:200). The tissues were then incubated with anti-rabbit IgG and biotinylated goat anti-rabbit antibody (Cat: ab64261, Abcam). The tissues were stained with diaminobenzidine (Cat# ab64261, Abcam).

### Primary and KGN Cell Culture

The establishment of the primary GCs line was performed as described previously (Liu and Hsueh, 1986; Reddy et al., 2008). Briefly, PD20 ovaries were dissected with residual fat and connective tissues removed. The ovaries were minced with a pair of dissection scissors before being incubated in 0.05% collagenase dissolved in McCoy’s 5A (Modified) medium (Cat: 16600-082, Gibco) supplemented with 4 mg/ml BSA, 100 units/ml penicillin, and 100 μg/ml streptomycin. Ethylenediaminetetraacetic acid (EDTA) was added to this reaction mixture to a final concentration of 40 mM followed by incubation at 37°C for 15 min. After removing the undigested tissues, the dispersed cells were washed twice with a McCoy’s 5A (Modified) medium and cultured in a 6-well plate in McCoy’s 5A (Modified) medium supplemented with 10% BSA to ensure the seeded cells reaching over 70% confluence. The cells were replaced with fresh McCoy’s 5A (Modified) medium 6 h later. VCD at different doses as well as the 50 ng/ml FSH as the positive control were added for 24 h. The VCD-exposed cells were harvested, and the cellular abundance of aromatase was measured by immunoblotting.

The human granulosa-like tumor KGN cell line (a gift from Professor Toshihiko Yanase of Fukuoka University in Japan to Center for Reproductive Medicine, Shandong University) was cultured in DMEM/F12 medium supplemented with 2% FBS (Gibco, United States) and 1% penicillin/streptomycin (Gibco, United States) in 5% CO_2_ at 37°C. The cells were plated in a 6-well plate at a density of 3–4 × 10^5^ cells per well with a cell passage number of 15–18. The cells were washed twice with PBS before VCD treatment. For the immunoblot analyses of aromatase expression levels, the cells were cultured in serum-free DMEM/F12 medium. FSH (50 ng/ml) was used as the positive control.

### Statistical Analyses

Statistical analyses were performed using SPSS 19.0 (IBM) and GraphPad Prism 7.04 (GraphPad Software, United States). All data are shown as the mean ± standard deviation (S.D.) of three independent biological replicates. Experimental data were analyzed using a one-way analysis of unpaired two-tailed Student’s *t*-tests. *P*-values of <0.05 were considered statistically significant.

## Results

### Very Short-Term VCD Exposure Promotes the Activation of Primordial Follicles by Transiently Activating the PI3K/Akt/mTOR Pathway, Whereas Extended Exposure Prevents Further Follicular Activation

**FIGURE 2:**
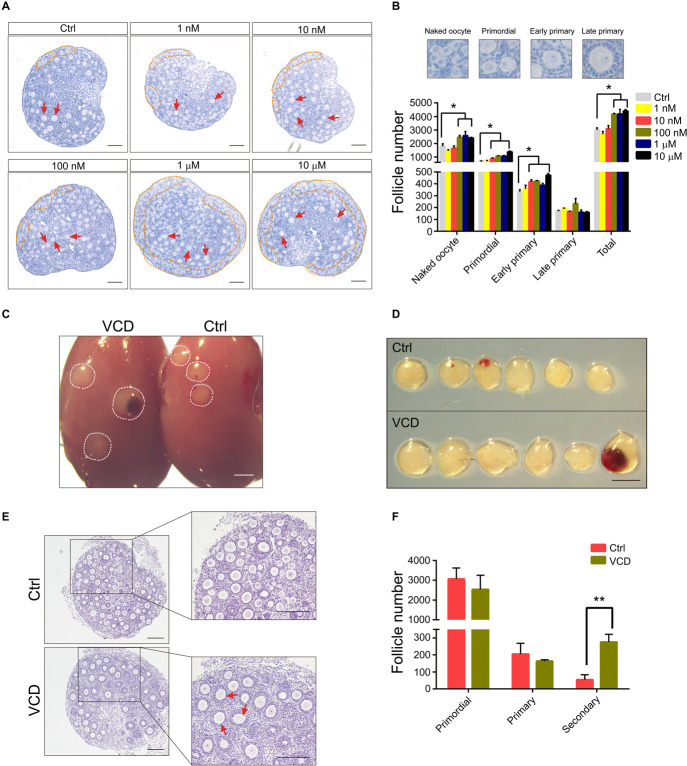
Effects of VCD exposure on the maintenance of primordial and primary follicle pools in PD2 mice. **(A)** Ovaries from PD2 mice were exposed to VCD (1 nM, 10 nM, 100 nM, 1 μM, and 10 μM) for 24 h and then incubated in VCD-free medium for 3 days, followed by histological examination with H&E staining. The primordial pool and individual primary follicles are highlighted by an orange broken line and red arrowheads, respectively. Scale bar = 100 μm. **(B)** Quantification of staged follicles (naked oocyte, primordial, early primary, and late primary) in VCD-exposed cultured ovaries from PD2 mice after a 4-day *in vitro* ovary culture. *P < 0.05 compared to aged-matched controls by Student’s *t*-test. The data are presented as the mean ± S.D. of *n* = 9 biological replicates per group. **(C)** Kidneys from a host mouse at 7 days post ovarian graft. Young adult (2-month) host mice were ovariectomized and given ovarian grafts from PD2 donor mice, followed by 3 days of VCD (80 mg/kg) starting 24 h after ovarian grafts. Scale bar = 400 μm. **(D)** The isolated ovaries from the kidney capsules of ovariectomized hosts 7 days after ovarian grafts. Scale bar = 400 μm. **(E)** Representative histological sections of VCD-exposed and control groups. Secondary follicles are marked by red arrowheads. Scale bar = 100 μm. **(F)** Quantification of primordial, primary, and secondary follicles in VCD-exposed ovaries. **P < 0.01 compared to the age-matched controls by Student’s *t*-test. The data are presented as the mean ± S.D. of *n* = 6 biological replicates per group.

We first investigated the effects of different doses of VCD exposure on the maintenance of the primordial and primary follicle pools in PD2 C57BL/6J mice using ovary culture *in vitro*. As shown in Figures 2A,B, exposure to VCD in concentrations ranging from 10 nM to 10 μM significantly increased the number of primordial and early primary follicles, as well as the total number of follicles compared to the controls (*P* < 0.05). In contrast, there was no difference in the number of late primary follicles between the VCD-exposed and control ovary cultures. These *in vitro* results suggest the possibility that VCD exposure might somehow protect primordial and primary follicles in PD2 mice from undergoing atretic degeneration.

Pursuing the supposition that VCD might protect primordial and primary follicles from atretic degeneration, we next conducted experiments following Li et al. (2010) based on ovarian grafts from PD2 mice onto the kidney of a 2-month-old mouse. Specifically, allograft-paired ovaries from a single PD2 donor mouse were separately transplanted into two 2-month-old ovariectomized host mice, one of which was exposed to VCD (via IP injection, 80 mg/kg daily for 3 days). Upon examination at post ovarian graft day 7, the transplanted ovaries of the VCD-exposed mice appeared larger and had significantly greater numbers of secondary follicles than the transplanted ovaries of the untreated control host mice (the mean number of secondary follicles was more than 5.2-fold higher in VCD-exposed mice; *P* < 0.05). No differences were observed in the numbers of primordial or primary follicles (Figures 2C–F).

The large increase that we observed in the number of secondary follicles upon VCD exposure, viewed alongside the lack of any change in the numbers of primordial and primary follicles, clearly suggests that an alternative fate (e.g., atretic degeneration) must be experienced by some population of follicles in the control mice. Thus, the results of our ovarian graft *in vivo* experiments are consistent with our *in vitro* cultured ovary experiments in supporting that VCD functions to prevent atretic degeneration. These findings also indicate that VCD might be involved in the primordial follicle activation (PFA) process. This was somewhat surprising given that VCD-induced follicular apoptosis is a well-documented mechanism that selectively destroys small preantral follicles in rodents (Chen et al., 2015).

**FIGURE 3:**
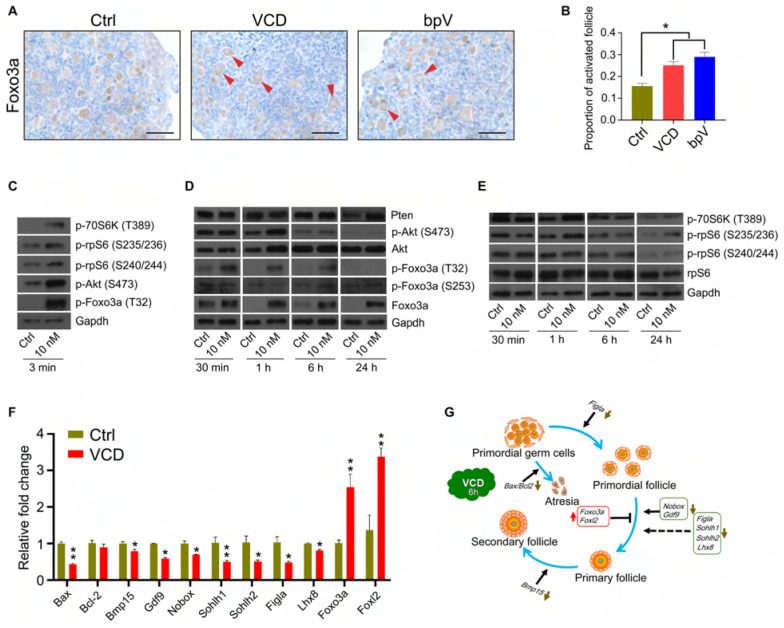
Very short-term VCD exposure transiently activates the PI3K-Akt-mTOR pathway activity, but extended exposure causes follicular development arrest. **(A)** Immunohistochemical staining of Foxo3a in cultured PD2 ovaries with or without VCD (10 nM) for 6 h. The Pten inhibitor *bpV* (100 μM) was added as a control. Scale bar = 50 μm. **(B)** Counting ratio of Foxo3a nuclear exclusion after 6 h of ovarian culture. * indicates *p* < 0.05; Student’s *t*-test. The data are presented as the mean ± S.D. of *n* = 6 biological replicates per group, and four tissue sections were counted per ovary. **(C)** Immunoblotting of phosphorylation levels for several proteins (P70S6k at Thr389, rpS6 at Ser235/236, rpS6 at Ser240/244, Foxo3a at Thr32, and Akt at Ser473) in cultured PD2 ovaries immediately after VCD exposure (3 min). Gapdh was used as the loading control. Representative blot images are shown, *n* = 3 biological replicates per group. **(D)** Immunoblotting of proteins of the PI3K-Akt-Foxo3a pathway (Foxo3a at Thr32, Foxo3a at Ser253, Foxo3a, Akt at Ser473, Akt, and Pten) in cultured PD2 ovaries exposed to 10 nM VCD for different times. Gapdh was used as the loading control. Representative blot images are shown, *n* = 3 biological replicates per group. **(E)** Immunoblotting of proteins of the mTOR pathway (P70S6k at Thr389, rpS6 at Ser235/236, rpS6 at Ser240/244, and rpS6) in cultured PD2 ovaries exposed to 10 nM VCD for different times. Gapdh was used as the loading control. Representative blot images are shown, *n* = 3 biological replicates per group. **(F)** qPCR expression analysis of genes related to early follicle development in cultured PD2 ovary samples treated with VCD (10 nM) for 6 h. * indicates *p* < 0.05 and ** indicates *p* < 0.01 by Student’s *t*-test, *n* = 3 biological replicates per group. **(G)** Diagram showing changes in the levels of selected transcription factors related to early follicle development after 6 h of VCD exposure.

We observed that 6 h of VCD exposure caused a significant increase in the nuclear exclusion of Foxo3a in primordial follicular oocytes, a finding similar to our observations after bisperoxovanadium (*bpV*) treatment (a Pten inhibitor) (Figures 3A,B). We further used cultured PD2 ovaries to investigate the effects of VCD exposure on the phosphatidylinositol 3 kinase (PI3K) and mammalian target of rapamycin (mTOR) signaling pathways. First, we noted that within 2–3 min of exposing the PD2 ovaries to VCD, Akt was activated (by phosphorylation at Ser473), and other extremely rapid signaling activations we detected within minutes included phosphorylation of Foxo3a at Thr32 and phosphorylation of the mTOR pathway component P70S6k at Thr389 (Figure 3C). The increase in Foxo3a phosphorylation at Thr32 was further elevated at 30 min, with this induction lasting for 6 h (Figure 3D). The phosphorylation levels of Akt at Ser473, P70S6k at Thr389, and rpS6 at Ser235/236 were also mildly elevated by VCD at 1 h (Figures 3D,E). These results indicate that VCD can activate primordial follicles very rapidly in ovarian cultures *in vitro*.

Interestingly, we also found that the protein level of Foxo3a increased from 1 h after VCD treatment, and found that the Pten protein level also increased after 24 h of VCD treatment (Figure 3D). These results suggest that a negative feedback control mechanism for follicle activation may also be activated by VCD. We used qPCR to measure the expression of several transcription factors known to function in early follicular development at 6 h after VCD treatment. In agreement with the immunoblotting results, the *Foxo3a* mRNA level was upregulated by 2.55-fold compared to the control group. We also detected an increase in the expression level of *Foxl2*, a gene known to be required for the maintenance of primordial follicles (Uda et al., 2004). The expression of *Figla*, *Nobox*, *Sohlh1*, *Sohlh2*, and *Lhx8* – which are genes that function in the transformation of primordial follicles into primary follicles (Lim and Choi, 2012) – was significantly downregulated by VCD treatment. Additionally, the attenuated expression levels we observed for *Gdf9* and *Bmp15* were consistent with the notion that PFA was inhibited at 6 h post VCD treatment (Figures 3F,G).

The role of VCD in inducing apoptosis in primordial follicles and primary follicles has been confirmed in many studies (Springer et al., 1996; Devine et al., 2004; Chen et al., 2015). However, it must be noted that the VCD concentrations used in previously reported studies were many times higher (∼1000×) than the concentration (10 nM) we used. It is also notable that previous studies all examined chronic VCD exposure in growth media, whereas we exposed ovaries to a low VCD concentration for 1 day followed by washout. Nevertheless, we conducted experiments to investigate VCD-induced follicular apoptosis, specifically by monitoring the effects of our short-term-VCD^low^ exposure on the expression of pro-and anti-apoptotic markers in cultured PD2 ovaries. We performed immunostaining and immunoblotting to measure the levels of the pro-apoptotic proteins caspase-3 (cleaved, the active form) and BCL-2-associated X (Bax) proteins and the antiapoptotic protein Bcl-2. In immunostained sections of PD12 mouse ovary tissue after 24 h of ovarian culture, the number of cells that stained positive for cleaved caspase-3 was significantly reduced in the VCD-treated ovarian sections compared to controls (Supplementary Figure S1A).

Next, to further investigate the effect of VCD on apoptosis in staged follicles, we isolated primordial, primary, and secondary follicles of PD12 mouse ovaries after 48 h of VCD exposure and measured the expression of *Bcl-2* and *Bax* using single-cell qPCR. We found that VCD exposure significantly increased the *Bcl-2* mRNA level in primordial follicles and did so in a dose-dependent manner. We also found that the *Bax/Bcl-2* level was significantly reduced in the VCD-exposed samples. Further, we found that VCD (1 μM) exposure caused a mild but significant decrease in *Bcl-2* levels in the primary and secondary follicles (Supplementary Figure S1B). Experiments using cultured PD2 ovarian tissue confirmed that the VCD-exposed group displayed a significant decrease in *Bax* expression compared to controls (Figure 3F). Supporting these qPCR-based findings, immunoblotting of extracts from cultured PD2 ovaries showed that 10 nM VCD exposure caused significant reductions in the Bax levels and caused an increase in the Bcl-2 protein level compared to the controls (Supplementary Figure S1C). Thus, our results support that low-concentration, short-term exposure to VCD somehow inhibits the expression of key genes of the Bax/Bcl-2 related apoptosis pathway.

### VCD Promotes Follicular Development *in vitro* and *in vivo* and Antagonizes Atretic Degeneration

**FIGURE 4:**
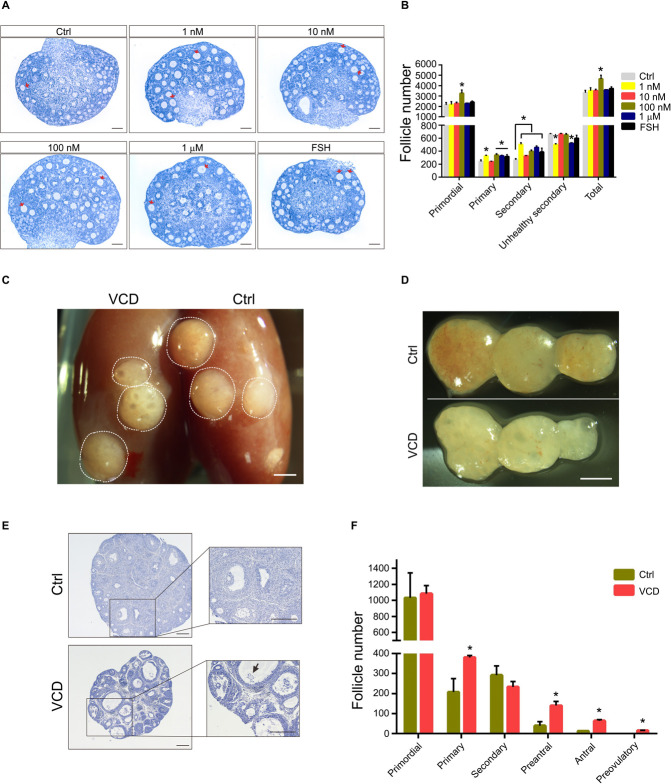
VCD promotes ovarian follicular development in PD12 mice after *in vitro* culture and ovarian transplantation. **(A)** Histological examination of H&E-stained ovaries from PD12 mice exposed to VCD (1 nM, 10 nM, 100 nM, and 1 μM) or FSH (50 ng/ml) for 4 days. The secondary follicles are marked by red arrowheads. Scale bar = 100 μm. **(B)** Quantification of staged follicles in VCD-exposed cultured PD12 ovaries after a 4-day *in vitro* ovary culture. *P < 0.05 compared to the age-matched controls by Student’s *t*-test. The data are presented as the mean ± S.D. of *n* = 9 biological replicates per group. **(C)** Kidneys from a host mouse at 14 days post ovarian graft. Young adult host mice were ovariectomized and given ovarian transplantation from PD12 donor mice, followed by 3 days of VCD (80 mg/kg) starting 24 h after ovarian grafts and 10 days of FSH (2 IU/day throughout the remainder of the experiment). Scale bar = 400 μm. **(D)** The isolated ovaries from the kidney capsules of ovariectomized hosts 14 days after ovarian grafts. Scale bar = 400 μm. **(E)** Representative histological sections of VCD-exposed and control groups. Preovulatory follicles are marked by black arrowheads. Scale bar = 200 μm. **(F)** Quantitation of primordial, primary, and secondary follicles in VCD-exposed ovaries. *P < 0.05 compared to the age-matched controls by Student’s *t*-test. The data are presented as the mean ± S.D. of *n* = 6 biological replicates per group.

We examined the effects of VCD exposure on the development of secondary follicles in PD12 mice using both *in vitro* ovary cultures and ovarian grafts. As shown in Figures 4A,B, after a 4-day VCD treatment follicle counting of serial ovarian sections from cultured ovaries revealed that VCD exposure significantly increased the number of secondary follicles at all of the tested concentrations (*P* < 0.05). VCD also significantly increased the number of primary follicles at all of the tested concentrations except 10 nM (*P* < 0.05). Additionally, VCD at 100 nM was able to increase the number of primordial follicles (*P* < 0.05).

The graft experiments were similar to the aforementioned protocol used with the PD2 ovary donors, but here the ovaries were from PD12 donors, ovary development was monitored at day 14 post ovarian graft, and FSH was administered (2 IU/day, IP injection) for the final 10 days of the experiment. Unlike controls, there were large follicles in the VCD-exposed ovaries (Figures 4C,D), and the average number of primary, preantral, antral, and preovulatory follicles in the VCD-treated ovaries were significantly elevated by 1.8-fold (*P* = 0.06), 3.5-fold (*P* = 0.024), 14.8-fold (*P* = 0.001), and 16.3-fold (*P* < 0.001) respectively, compared to the control groups. In contrast, we observed no differences in the numbers of primordial or secondary follicles (Figures 4E,F).

We subsequently conducted follicular development experiments with PD12 mice in which we examined dose-response-time relationships upon exposure to two substantially different concentrations of VCD (20 mg/kg or 160 mg/kg) via IP injection for 5, 10, or 15 days. As shown in Supplementary Figures S2A–D, VCD dose-dependently increased the number of early antral and antral follicles for the 5-day and 10-day treatment but did not affect the number of primordial, primary, or secondary follicles. Exposure to 20 mg/kg VCD for 15 days increased the number of primordial, primary, secondary, early antral, antral, and preovulatory follicles, whereas 160 mg/kg VCD for 15 days reduced the number of primordial and primary follicles compared to the control groups. Thus our results are congruent with previously reported findings that high-concentration, long-term exposure to VCD can induce follicular apoptosis (Rappaport and Fraser, 1977; Devine et al., 2001), but they also show that low-concentration, short-term exposure to VCD can promote follicular development.

### VCD Promotes Superovulation in Middle-Aged Mice

**FIGURE 5:**
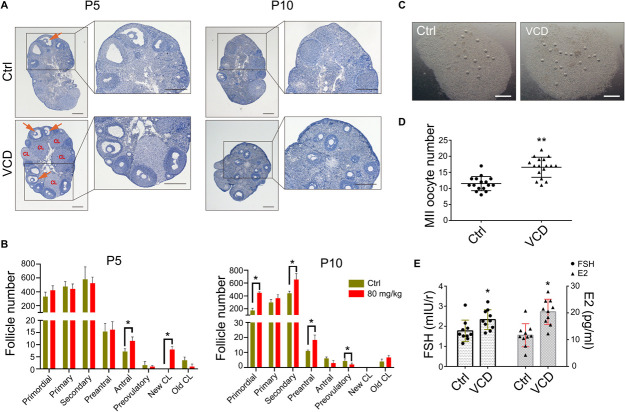
Effects of VCD exposure on the superovulatory response in middle-aged mice. **(A)** Histological examination of hematoxylin-stained ovaries from 10 to 12-month-old mice (“middle-aged”) given IP injection of VCD (80 mg/kg) daily for 5 or 10 days. The preovulatory follicles are marked by orange arrowheads. The newly formed corpus luteum are marked with the letters *CL*. Scale bar = 200 μm. **(B)** Quantification of staged follicles [primordial, primary, secondary, preantral, antral, preovulatory, newly formed corpus luteum (new CL), old corpus luteum (old CL)] in VCD-exposed ovaries from middle-aged mice given the 5 or 10 days IP injection of VCD. *P < 0.05 compared to aged-matched controls by Student’s *t*-test. The data are presented as the mean ± S.D., *n* = 6 biological replicates per group. **(C)** After a daily IP injections of VCD (80 mg/kg) for 5 days, mice were injected IP with 5 IU of PMSG for 48 h before injection of 10 IU of human chorionic gonadotropin (hCG). Sixteen hours after the hCG injection, cumulus-oocyte complexes were collected from the fallopian tubes. Scale bar = 500 μm. **(D)** Quantification of MII oocytes in VCD-treated (80 mg/kg, IP) and control mice. **P < 0.01 compared to the age-matched controls by Student’s *t*-test. The data are presented as the mean ± S.D., *n* = 16–17 biological replicates per group. **(E)** Quantification of FSH and E2 serum levels in middle-aged mice exposed to VCD (80 mg/kg/day, IP) for 5 days. *P < 0.05 compared to the aged-matched controls. The data are presented as the mean ± S.D. of *n* = 10 biological replicates per group.

Ovarian aging is associated with a decline in the number and quality of oocytes, with studies reporting increases in the percentage of abnormal/degenerated oocytes (Tarin et al., 2001). The size of the primordial follicle pool is also correlated with the number of follicles able to enter the growing pool (Hirshfield, 1994). Given our *in vitro* and *in vivo* findings that VCD exposure promotes follicular development, we investigated whether short-term VCD can improve the function of ovaries in middle-aged (10–12-month-old) mice and perhaps increase superovulation efficiency (i.e., the drug-induced production of multiple oocytes for use in assisted reproductive technologies such as IVF). We exposed 10–12-month-old mice to 80 mg/kg VCD for 5 or 10 days and found that 5-day VCD exposure significantly increased the number of antral follicles and corpus luteum. VCD exposure for 10 days increased the number of secondary follicles; however, the number of preovulatory follicles decreased with the 10-day exposure. Interestingly, during this period we also observed that the number of primordial follicles in the VCD-exposed mice was higher than that in control mice (Figures 5A,B).

We next examined whether 5-day VCD exposure could promote superovulation in the middle-aged mice. Figures 5C,D shows that the number of MII oocyte in cumulus cell-oocyte complexes from oviducts at 16 h post-hCG administration increased by 1.4-fold (*P* < 0.0001) compared to control mice. Additionally, analysis of serum from 5-day VCD-exposed mice revealed a 1.3-fold (*P* = 0.025) increase in the FSH concentration and a 1.8-fold (*P* = 0.0004) increase in the E2 concentration compared to the age-matched controls (Figure 5E). The trend of VCD in promoting ovulation was verified in young-adult mice (Supplementary Figure S3A). We also found that VCD exposure for 5–10 days disrupted the estrous cycle in mice. About 35% of the VCD-exposed mice exhibited proestrus prolongation, and about 40% of these mice displayed shorter estrus intervals (estrus-estrus period) (Supplementary Figure S3B). This VCD-induced superovulation in young adult and middle-aged mice was consistent with our results showing that VCD could promote follicular development.

### VCD Stimulates Estrogen Production by Promoting Aromatase Expression

**FIGURE 6:**
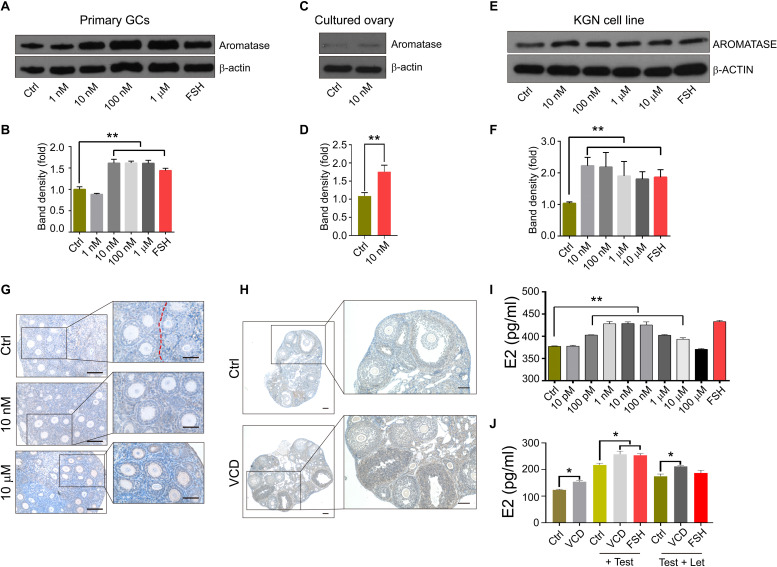
The aromatase-promoting effects of VCD. **(A)** Immunoblotting with antibodies against aromatase and β-actin of extracts from primary ovarian GCs of PD20 mice treated with VCD (1 nM, 10 nM, 100 nM, or 1 μM) or FSH (50 ng/ml) for 24 h. **(B)** Quantitation of **(A)** using the gray value detection module of ImageJ; ** indicates *p* ¡ 0.01 compared to untreated controls by Student’s *t*-test. The data are presented as the mean ± S.D. of *n* = 3 biological replicates per group. **(C,D)** The β-actin-normalized protein expression levels of aromatase in cultured PD12 ovaries exposed to 10 nM VCD for 24 h were analyzed using immunoblotting, with quantification using the ImageJ software. Representative blot images are shown. ** indicates *p* ¡ 0.01 compared to the drug-paired controls by Student’s *t*-test. The data are presented as mean ± S.D. of *n* = 3 biological replicates per group. **(E,F)** The human granulosa-like tumor KGN cell line cultured in FBS-free DMEM/F12 medium exposed to VCD at 10 nM, 100 nM, 1 μM, and 10 μM or FSH (50 ng/ml) for 24 h was analyzed using immunoblotting, with quantification using the ImageJ software. Representative blot images are shown. ** indicates *p* ¡ 0.01 compared to the drug-paired controls by Student’s *t*-test. The data are presented as the mean ± S.D. of *n* = 3 biological replicates per group. **(G)** Histological examination of ovarian aromatase-stained PD12 mice exposed to VCD at 10 nM or 10 μM for 24 h *in vitro*. Scale bar = 100 μm. **(H)** Histological examination of ovarian aromatase-stained PD28 mice IP injected with VCD (80 mg/kg) once each day for 5 days. Scale bar = 100 μm. **(I)** Exposure to VCD at 10 pM, 100 pM, 1 nM, 10 nM, 100 nM, 1 μM, 10 μM, or 100 μM (or PBS control) for 24 h. The estrogen levels in the KGN cell culture supernatants were measured at 24 h using ELISA. The data are presented as the mean ± S.D. of *n* = 3 biological replicates per group. **(J)** VCD exposure for 24 h (with or without the aromatase substrate testosterone at 10 nM), followed by ELISA-based measurement of estrogen levels at 24 h in the PD12 ovarian culture supernatants. PBS was the negative control and FSH (50 ng/ml) was the positive control, letrozole (Let) is used as aromatase inhibitor. The data are presented as the mean ± S.D. of *n* = 3 biological replicates per group.

Given our finding that VCD administration elevates E2 concentrations in the serum of middle-aged mice (Figure 5E), and considering the known role of aromatase in the biosynthesis of estrogen by ovarian GCs (Jia and Hsueh, 1986; Liu and Hsueh, 1986), we investigated the effects of VCD exposure on aromatase expression in multiple cell types, including primary mouse ovarian GCs and *in vitro* cultured PD12 mouse ovaries. VCD exposure for 24 h (doses from 10 nM to 1 μM) significantly promoted aromatase protein expression in cultured primary ovarian GCs from PD20 mice (Figures 6A,B).

We also performed immunostaining to examine the aromatase expression in VCD-exposed cultured PD12 ovaries (*in vitro*) and PD28 mouse ovaries (*in vivo*) 5 days after IP injection. For ovaries of PD12 mice cultured *in vitro*, we found that the intensity of the staining signal for aromatase was elevated in the VCD-exposed (10 nM and 10 μM) ovarian sections compared to controls, and it is notable that we detected barely any aromatase expression in the surrounding GCs with abnormal morphology (Figure 6G). Immunoblotting showed that 10 nM VCD exposure for 24 h increased the aromatase expression levels in cultured PD12 ovaries by 1.7-fold (*P* < 0.0001) (Figures 6C,D). In the ovary of PD28 mice after 5-day VCD injection, we found that the intensity of the staining signal for aromatase in all stages of follicles was higher than that of controls (Figure 6H).

Additionally, VCD exposure promoted the protein expression of aromatase in the steroidogenic human granulosa-like tumor KGN cell line (Figures 6E,F), and VCD exposure (from 100 pM to 10 μM) also significantly enhanced the E2 secretion from these cells. Note that the 100 μM VCD exposure resulted in significantly reduced E2 secretion levels compared to untreated control KGN cells, suggesting that high-dose VCD may inhibit E2 secretion (Figure 6I). We also examined the effect of 10 nM VCD exposure on the E2 secretion from cultured PD12 ovaries, and regardless of the presence/absence of the aromatase substrate testosterone in the ovarian culture medium, the E2 level was significantly elevated in the medium of VCD-treated ovaries as compared to untreated control cultures (Figure 6J). Additionally, we found that 10 nM VCD exposure could partially attenuate the effects of letrozole, an aromatase inhibitor known to inhibit E2 secretion (Figure 6J). Collectively, these results show that exposure to low concentrations of the known endocrine disruptor compound VCD promotes E2 secretion from GCs by inducing aromatase expression, potentially helping to explain the observed impacts of VCD in promoting folliculogenesis.

## Discussion

Phosphatidylinositol 3 kinase signaling has been repeatedly shown to be essential for the awakening of the dormant primordial follicle pool, the recruitment of primordial follicles, oocyte survival, and follicular maturation (Kim and Kurita, 2018). The mTORC1 signaling pathway coordinates with the PI3K pathway to control the activation process of primordial follicles (Reddy et al., 2009; Adhikari et al., 2010), and Pten and Foxo3a act as suppressors during PFA (Castrillon et al., 2003; Reddy et al., 2008). The non-phosphorylated form of Foxo3a is transcriptionally active in nuclei, and active Foxo3 in oocytes helps to preserve the ovarian reserve in mice (Pelosi et al., 2013). We found that VCD could quickly activate the PI3K-Akt pathway and phosphorylate Foxo3a at Thr32, which was accompanied by activation of mTOR pathway components (Figure 3C), and this implicated the activation of PFA-related pathways in the VCD-mediated promotion of primary follicles in cultured PD2 mouse ovaries (Figures 2A,B). Previous studies have reported that VCD can promote recruitment from primordia to small primary follicles via the PI3K pathway and have shown that such recruitment can be attenuated by the PI3 kinase inhibitor LY294002 (Keating et al., 2009). Another study demonstrated that adverse effects from long-term high-dose VCD treatment on mouse ovaries are mediated by the Rictor/mTORC2/Akt/Foxo3a pathway (Chen et al., 2015).

However, our results establish that *Foxo3a* expression and Foxo3a accumulation in the VCD-exposed group was upregulated soon after VCD exposure (Figures 3D,F). We speculate that VCD-induced expression of *Foxo3a* may functionally contribute to the observed impact of VCD in maintaining the primordial follicle pool. Supporting this, our observations after 6 h of VCD exposure revealed reductions in the expression levels of several transcription factors known to function in promoting early follicular development (Figure 3F).

**FIGURE 7:**
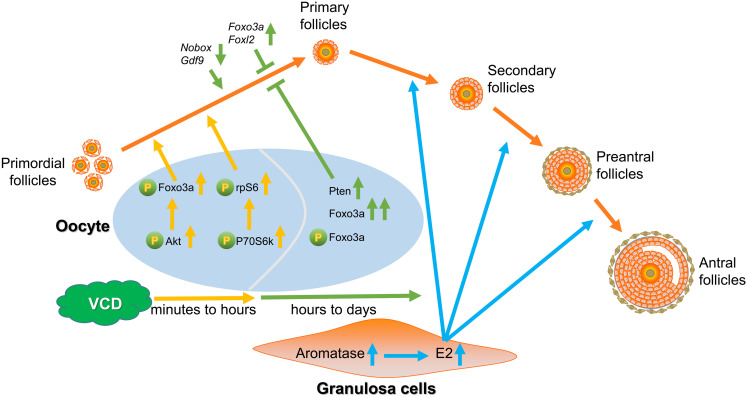
Model for the dichotomous effects of VCD on follicular development. Short-exposure VCD treatment transiently activates key proteins in the PI3K-Akt pathway and mTOR pathway, with the phosphorylation levels of different phosphorylation sites of Akt, Foxo3a, P70S6k, and rpS6 increasing to varying degrees, thereby activating a portion of the primordial follicle pool. Upon extended VCD exposure (i.e., exposure time exceeding 6 h), the expression of negative regulatory factors for follicle activation increases (*Foxo3a* and *Foxl2*) but the expression decreases for transcription factors that promote early follicular development and PFA (e.g., *Nobox*, *Gdf9*, *Figla*, *Sohlh1*, and *Sohlh2*). Additionally, the Pten level is elevated after 24 h of VCD exposure. Thus, starting from around hour 6 h of VCD exposure, a “braking mechanism” is activated that inhibits the activation or further development of resting primordial follicles. VCD induces the expression of aromatase in secondary follicles, thereby promoting further development of secondary follicles. VCD therefore exhibits dichotomous effects based on dosage and treatment time and has specific effects on follicles at particular development stages.

From experimental results of ovarian culture and ovarian transplantation, we found that VCD dosing from 1 nM to 1 μM for 4 days broadly elevated the number of primordial, primary, and secondary follicles in cultured ovaries (Figures 4A,B) and transplanted ovaries (Figures 4E,F), whereas exposure of 160 mg/kg VCD for 15 days promoted the process of occlusion apoptosis in primordial follicles and primary follicles (Supplementary Figure S2D). Considered alongside our observations of the impacts of VCD on resting follicles, our results with growing-phase follicles clearly support that VCD causes dichotomous effects dependent on dose, time, and follicle development stage. That is, VCD can both maintain quiescent follicles and promote the development of growing follicles. The dichotomous effect of VCD on follicular development is shown in Figure 7.

Prior studies have indicated that perimenopausal women with a diminished supply of FSH-responsive follicles experience intermittent ovulation or even chronic anovulation during this transition due to hormonal imbalance and menstrual irregularity (Nicula and Costin, 2015). Given our findings from mice that VCD (within a given concentration range) can function to promote oocyte maturation and to promote ovulation independent of FSH, it is of biological and clinical relevance to further examine whether VCD *per se* may be able to improve ovulatory responses and fertility in perimenopausal women.

Polycystic ovary syndrome (PCOS), characterized by hirsutism, hyperandrogenism, and polycystic ovaries, is the most prevalent endocrine disorder of reproductive-aged women worldwide (Franks et al., 2008). On the one hand, previous studies have indicated that the number of growing follicles are significantly higher in PCOS ovaries, but the follicles are developmentally arrested at the mid-antral stage, possibly due to premature luteinization (Chang and Cook-Andersen, 2013). On the other hand, women suffering from POI experience early exhaustion of ovarian follicles (Kovanci and Schutt, 2015). However, residual resting primordial follicles remain (Hsueh et al., 2015). Recently, Kawamura et al. (2016) developed a novel *in vitro* activation treatment for POI patients. It has been proposed that activation of the dormant primordial follicular pool could provide a new source of oocytes for fertilization in both PCOS and POI patients (Chang and Cook-Andersen, 2013; Kawamura et al., 2016). Our results suggest that VCD may be useful for protecting the primordial/primary pool and for promoting follicular maturation and ovulation. It would thus be of clinical significance to examine the possibility of the therapeutic use of VCD to improve FOR and fertility in PCOS and POI patients.

The hormone-like effects of VCD include its enhancement of aromatase expression in primary GCs, cultured ovaries, and KGN cells, which in turn promotes E2 secretion, so it is possible that VCD may exert an FSH-like function for increasing aromatase expression and E2 secretion from GCs to promote follicular maturation. This would be consistent with a prior study reporting that E2 (0.1 mg/kg) can protect small preantral follicles from VCD-induced atresia due to apoptosis (80 mg/kg/day for 15 days, IP), thereby promoting folliculogenesis (Thompson et al., 2002).

Our molecular docking analysis of VCD revealed its interaction with aromatase (CYP19A1), and VCD is predicted to form a hydrogen bond with Met374 and hydrophobic contacts with Val370, Leu477, and lle133 (Supplementary Figure S4). It is also worth reiterating our finding that VCD elevates serum FSH concentrations in middle-aged mice with DOR (Figure 5E), thus implying that VCD may act on the hypothalamic-pituitary-ovary axis to regulate the hormonal milieu for oocyte maturation and ovulation. Moreover, while we did examine mice of different ages in the study, it seems likely that experiments using an ovarian failure animal model would be informative in future investigations to help delineate VCD impacts on the specific follicle populations that are found in various subfertility disorders.

## Conclusion

Our study shows that very short-term VCD exposure can activate a portion of the primordial follicle pool by transiently activating the PI3K-akt and mTOR pathways, and it shows that extended VCD exposure then initiates a braking mechanism that halts follicle activation, thereby preventing the activation of the remaining primordial follicles of the pool. We provide evidence showing that VCD broadly promotes follicular development and maturation by increasing E2 secretion resulting from elevated aromatase expression in GCs. Excitingly, we found that short-term VCD^low^ exposure profoundly enhances superovulatory responses in middle-aged mice. Thus, our work substantially deepens our understanding of the apparent dose- and duration-dependent hormone-like opposing effects of VCD^low^ on follicular development and ovulation induction. Looking forward, we plan to further investigate the mechanisms that cause the differential effects upon extended VCD exposure. We also plan to explore how different VCD concentrations impact resting follicles and growing follicles, and these studies may facilitate exploitation of VCD’s positive effects on follicular development in order to improve fertility.

## Data Availability Statement

All datasets presented in this study are included in the article/Supplementary Material.

## Ethics Statement

The animal study was reviewed and approved by the University Animal Experiments Ethics Committee of the CUHK and Ethics Review Board of the Center for Reproductive Medicine of Shandong University.

## Author Contributions

JM, GL, and WC designed the experiments. LC undertook most of the experimental work and participated in the literature search. XS helped with cell culture. HBL and YL assisted with animal experiments and HE staining. HJL provided bioinformatics support. YD and ZX helped with data analysis. JM, Z-JC, and WC conceived the study. LC, CL, and WW interpreted the data and wrote the manuscript with the assistance of other authors. All authors contributed to the article and approved the submitted version.

## Conflict of Interest

The authors declare that the research was conducted in the absence of any commercial or financial relationships that could be construed as a potential conflict of interest.

## Supplementary Material

FIGURE S1 VCD short-term treatment reduces follicular apoptosis. **(A)** Immunohistochemical staining of cleaved caspase3 in cultured PD2 ovaries with or without VCD (10 nM, 10 μM) for 24 h. Yellow arrows indicate cleaved caspase-3-positive cells. PD2 ovary scale bar = 50 μm, PD12 ovary scale bar = 100 μm. **(B)** Quantification by staged single-oocyte qPCR of the mRNA expression of *Bcl-2* and *Bax* in isolated primordial (20 μm), primary (40–50 μm), and secondary follicles (70 μm) derived from PD12 ovaries cultured with VCD (10 nM, 1 μM) and FSH (50 ng/ml) for 48 h. *indicates *p* < 0.05 and **indicates *p* < 0.01 by Student’s *t*-test, *n* = 3 biological replicates per group. **(C)** The β-actin-normalized protein expression levels of Bax and Bcl-2 in cultured PD2 ovaries exposed to 10 nM VCD for 2 days were analyzed using immunoblotting.

FIGURE S2 VCD broadly promotes follicular development *in vivo.* **(A)** Histological examination of H&E-stained ovaries from PD12 mice given IP injection of VCD (20 mg/kg or 160 mg/kg) daily for 5, 10, or 15 days. Preantral and antral follicles are marked by red and yellow arrowheads, respectively. Scale bar = 100 μm. **(B–D)** Quantification of staged follicles in VCD-exposed PD12 ovaries for 5, 10, and 15 days. ^∗^ indicates *p* < 0.05 compared to aged-matched controls by ANOVA. The data are presented as the mean ± S.D. of *n* = 3–6 biological replicates per group.

FIGURE S3 Impacts of VCD exposure on follicular development and ovulation in young mice. **(A)** After daily IP injections of VCD (80 mg/kg) for 5 days, 2-month-old young adult mice were injected IP with 5 IU of PMSG for 48 h before injection of 10 IU of human chorionic gonadotropin (hCG). Sixteen hours after the hCG injection, cumulus-oocyte complexes were collected from the fallopian tubes. Scale bar = 500 μm. Quantification of MII-stage oocytes in VCD-exposed 2-month-old mouse ovaries. *indicates *p* < 0.05 by Student’s *t*-test. The data are presented as the mean ± S.D. of *n* = 15 biological replicates per group. **(B)** With or without 10 days of IP injection of VCD (160 mg/kg) in mice, the estrous cycle at different time points was detected by vaginal lavage. **(C)** Two-month-old mice with or without 10 days of IP injection of VCD (80 mg/kg). Scale bar = 500 μm. **(D)** Histological examination of H&E-stained ovaries from 2-month-old mice exposed to VCD (80 mg/kg) via IP for 5 or 10 days. The mice were sacrificed after the final injection. Scale bar = 100 μm.

FIGURE S4 Computational predictions of VCD-aromatase binding. **(A)** Cytochrome P450 19A1 is predicted to be the primary target of VCD. **(B)** Twelve known small-molecule binders of Cytochrome P450 19A1, as well as a comparison of the structural similarity of these 12 compounds to VCD based on ChEMBL ID, SMILES, and 2D information. **(C)** Molecular structures of the top three known binders of Cytochrome P450 19A1. **(D)** 3D visualization of the putative binding conformation of VCD with aromatase (left), and 2D visualization of the putative binding interactions of VCD with aromatase (right). **(E)** 3D visualization of the putative binding conformation of VCD overlaid with the crystal binding conformation of the known substrate androstenedione.

## References

[B1] Adhikari D. Liu K. (2009). Molecular mechanisms underlying the activation of mammalian primordial follicles. *Endocr. Rev.* 30 438–464. 10.1210/er.2008-0048 19589950

[B2] Adhikari D. Zheng W. Shen Y. Gorre N. Hamalainen T. Cooney A. J. (2010). Tsc/mTORC1 signaling in oocytes governs the quiescence and activation of primordial follicles. *Hum. Mol. Genet.* 19 397–410. 10.1093/hmg/ddp483 19843540 PMC2798719

[B3] Appt S. E. Kaplan J. R. Clarkson T. B. Cline J. M. Christian P. J. Hoyer P. B. (2006). Destruction of primordial ovarian follicles in adult cynomolgus macaques after exposure to 4-vinylcyclohexene diepoxide: a nonhuman primate model of the menopausal transition. *Fertil. Steril.* 86(4 Suppl.) 1210–1216. 10.1016/j.fertnstert.2006.05.004 16952357

[B4] Bhattacharya P. Keating A. F. (2012). Impact of environmental exposures on ovarian function and role of xenobiotic metabolism during ovotoxicity. *Toxicol. Appl. Pharmacol.* 261 227–235. 10.1016/j.taap.2012.04.009 22531813 PMC3359424

[B5] Buckler H. (2005). The menopause transition: endocrine changes and clinical symptoms. *J. Br. Menopause Soc.* 11 61–65. 10.1258/136218005775544525 15970017

[B6] Castrillon D. H. Miao L. Kollipara R. Horner J. W. DePinho R. A. (2003). Suppression of ovarian follicle activation in mice by the transcription factor Foxo3a. *Science* 301 215–218. 10.1126/science.1086336 12855809

[B7] Chang R. J. Cook-Andersen H. (2013). Disordered follicle development. *Mol. Cell. Endocrinol.* 373 51–60. 10.1016/j.mce.2012.07.011 22874072 PMC3727408

[B8] Chen Z. Kang X. Wang L. Dong H. Wang C. Xiong Z. (2015). Rictor/mTORC2 pathway in oocytes regulates folliculogenesis, and its inactivation causes premature ovarian failure. *J. Biol. Chem.* 290 6387–6396. 10.1074/jbc.m114.605261 25564616 PMC4358274

[B9] de Mello Bianchi P. H. Serafini P. Monteiro da Rocha A. Assad Hassun P. Alves da Motta E. L. Sampaio Baruselli P. (2010). Review: follicular waves in the human ovary: a new physiological paradigm for novel ovarian stimulation protocols. *Reprod. Sci.* 17 1067–1076. Sampaio Baruselli P D:NOPMID 10.1177/1933719110366483 20439948

[B10] Devine P. J. Sipes I. G. Hoyer P. B. (2001). Effect of 4-vinylcyclohexene diepoxide dosing in rats on GSH levels in liver and ovaries. *Toxicol. Sci.* 62 315–320. 10.1093/toxsci/62.2.315 11452144

[B11] Devine P. J. Sipes I. G. Hoyer P. B. (2004). Initiation of delayed ovotoxicity by in vitro and in vivo exposures of rat ovaries to 4-vinylcyclohexene diepoxide. *Reprod. Toxicol.* 19 71–77. 10.1016/j.reprotox.2004.06.002 15336714

[B12] Devine P. J. Sipes I. G. Skinner M. K. Hoyer P. B. (2002). Characterization of a rat in vitro ovarian culture system to study the ovarian toxicant 4-vinylcyclohexene diepoxide. *Toxicol. Appl. Pharmacol.* 184 107–115. 10.1006/taap.2002.9502 12408955

[B13] Fernandez S. M. Keating A. F. Christian P. J. Sen N. Hoying J. B. Brooks H. L. (2008). Involvement of the KIT/KITL signaling pathway in 4-vinylcyclohexene diepoxide-induced ovarian follicle loss in rats. *Biol. Reprod.* 79 318–327. 10.1095/biolreprod.108.067744 18448842 PMC2714991

[B14] Fisher C. R. Graves K. H. Parlow A. F. Simpson E. R. (1998). Characterization of mice deficient in aromatase (ArKO) because of targeted disruption of the cyp19 gene. *Proc. Natl. Acad. Sci. U.S.A.* 95 6965–6970. 10.1073/pnas.95.12.6965 9618522 PMC22703

[B15] Franks S. Stark J. Hardy K. (2008). Follicle dynamics and anovulation in polycystic ovary syndrome. *Hum. Reprod. Update* 14 367–378. 10.1093/humupd/dmn015 18499708

[B16] Hirshfield A. N. (1994). Relationship between the supply of primordial follicles and the onset of follicular growth in rats. *Biol. Reprod.* 50 421–428. 10.1095/biolreprod50.2.421 8142559

[B17] Hsueh A. J. Kawamura K. Cheng Y. Fauser B. C. (2015). Intraovarian control of early folliculogenesis. *Endocr. Rev.* 36 1–24. 10.1210/er.2014-1020 25202833 PMC4309737

[B18] Hu X. Christian P. Sipes I. G. Hoyer P. B. (2001). Expression and redistribution of cellular Bad, Bax, and Bcl-X(L) protein is associated with VCD-induced ovotoxicity in rats. *Biol. Reprod.* 65 1489–1495. 10.1095/biolreprod65.5.1489 11673266

[B19] Hu X. Roberts J. R. Apopa P. L. Kan Y. W. Ma Q. (2006). Accelerated ovarian failure induced by 4-vinyl cyclohexene diepoxide in Nrf2 null mice. *Mol. Cell. Biol.* 26 940–954. 16428448 10.1128/MCB.26.3.940-954.2006 PMC1347017

[B20] Jia X. C. Hsueh A. J. (1986). Granulosa cell aromatase bioassay for follicle-stimulating hormone: validation and application of the method. *Endocrinology* 119 1570–1577. 10.1210/endo-119-4-1570 2428600

[B21] Kappeler C. J. Hoyer P. B. (2012). 4-vinylcyclohexene diepoxide: a model chemical for ovotoxicity. *Syst. Biol. Reprod. Med.* 58 57–62. 10.3109/19396368.2011.648820 22239082 PMC3307534

[B22] Kawamura K. Kawamura N. Hsueh A. J. (2016). Activation of dormant follicles: a new treatment for premature ovarian failure? *Curr. Opin. Obstet. Gynecol.* 28 217–222. 10.1097/gco.0000000000000268 27022685 PMC5536116

[B23] Keating A. F. Mark J. C. Sen N. Sipes I. G. Hoyer P. B. (2009). Effect of phosphatidylinositol-3 kinase inhibition on ovotoxicity caused by 4-vinylcyclohexene diepoxide and 7, 12-dimethylbenz[a]anthracene in neonatal rat ovaries. *Toxicol. Appl. Pharmacol.* 241 127–134. 10.1016/j.taap.2009.08.012 19695275 PMC2783260

[B24] Kim S. Y. Kurita T. (2018). New insights into the role of phosphoinositide 3-kinase activity in the physiology of immature oocytes: lessons from recent mouse model studies. *Eur. Med. J. Reprod. Health* 3 119–125. 30245862 PMC6147255

[B25] Kovanci E. Schutt A. K. (2015). Premature ovarian failure: clinical presentation and treatment. *Obstet. Gynecol. Clin. North Am.* 42 153–161. 25681846 10.1016/j.ogc.2014.10.004

[B26] Li J. Kawamura K. Cheng Y. Liu S. Klein C. Liu S. (2010). Activation of dormant ovarian follicles to generate mature eggs. *Proc. Natl. Acad. Sci. U.S.A.* 107 10280–10284. 10.1073/pnas.1001198107 20479243 PMC2890455

[B27] Lim E. J. Choi Y. (2012). Transcription factors in the maintenance and survival of primordial follicles. *Clin. Exp. Reprod. Med.* 39 127–131. 23346521 10.5653/cerm.2012.39.4.127 PMC3548069

[B28] Liu Y. X. Hsueh A. J. (1986). Synergism between granulosa and theca-interstitial cells in estrogen biosynthesis by gonadotropin-treated rat ovaries: studies on the two-cell, two-gonadotropin hypothesis using steroid antisera. *Biol. Reprod.* 35 27–36. 10.1095/biolreprod35.1.27 3091103

[B29] Naz R. K. (2005). *Endocrine Disruptors: Effects on Male and Female Reproductive Systems*, 2nd Edn Boca Raton, FL: CRC Press.

[B30] Nicula R. Costin N. (2015). Management of endometrial modifications in perimenopausal women. *Clujul Med.* 88 101–110. 10.15386/cjmed-421 26528056 PMC4576794

[B31] Pelosi E. Omari S. Michel M. Ding J. Amano T. Forabosco A. (2013). Constitutively active Foxo3 in oocytes preserves ovarian reserve in mice. *Nat. Commun.* 4:1843. 10.1038/ncomms2861 PMC4504230 23673628

[B32] Rappaport S. M. Fraser D. A. (1977). Air sampling and analysis in a rubber vulcanization area. *Am. Ind. Hyg. Assoc. J.* 38 205–210. 10.1080/0002889778507601 868759

[B33] Reddy P. Adhikari D. Zheng W. Liang S. Hamalainen T. Tohonen V. (2009). PDK1 signaling in oocytes controls reproductive aging and lifespan by manipulating the survival of primordial follicles. *Hum. Mol. Genet.* 18 2813–2824. 10.1093/hmg/ddp217 19423553

[B34] Reddy P. Liu L. Adhikari D. Jagarlamudi K. Rajareddy S. Shen Y. (2008). Oocyte-specific deletion of Pten causes premature activation of the primordial follicle pool. *Science* 319 611–613. 10.1126/science.1152257 18239123

[B35] Springer L. N. Tilly J. L. Sipes I. G. Hoyer P. B. (1996). Enhanced expression of bax in small preantral follicles during 4-vinylcyclohexene diepoxide-induced ovotoxicity in the rat. *Toxicol. Appl. Pharmacol.* 139 402–410. 10.1006/taap.1996.0181 8806858

[B36] Tarin J. J. Perez-Albala S. Cano A. (2001). Cellular and morphological traits of oocytes retrieved from aging mice after exogenous ovarian stimulation. *Biol. Reprod.* 65 141–150. 10.1095/biolreprod65.1.141 11420234

[B37] Thompson K. E. Sipes I. G. Greenstein B. D. Hoyer P. B. (2002). 17beta-estradiol affords protection against 4-vinylcyclohexene diepoxide-induced ovarian follicle loss in Fischer-344 rats. *Endocrinology* 143 1058–1065. 10.1210/endo.143.3.8665 11861533

[B38] Torrealday S. Kodaman P. Pal L. (2017). Premature ovarian insufficiency – an update on recent advances in understanding and management. *F1000Res* 6:2069. 10.12688/f1000research.11948.1 29225794 PMC5710309

[B39] Uda M. Ottolenghi C. Crisponi L. Garcia J. E. Deiana M. Kimber W. (2004). Foxl2 disruption causes mouse ovarian failure by pervasive blockage of follicle development. *Hum. Mol. Genet.* 13 1171–1181. 10.1093/hmg/ddh124 15056605

[B40] Vabre P. Gatimel N. Moreau J. Gayrard V. Picard-Hagen N. Parinaud J. (2017). Environmental pollutants, a possible etiology for premature ovarian insufficiency: a narrative review of animal and human data. *Environ. Health* 16 37. 10.1186/s12940-017-0242-4 PMC5384040 28388912

