# Corrigendum: Hormone-Like Effects of 4-Vinylcyclohexene Diepoxide on Follicular Development

**DOI:** 10.3389/fcell.2020.607067

**Published:** 2020-12-03

**Authors:** Lian Bao Cao, Hong Bin Liu, Gang Lu, Yue Lv, Chi Kwan Leung, Yan Zhi Du, Wu Ming Wang, Zhi Qiang Xiong, Xian Wei Su, Hong Jian Li, Zi-Jiang Chen, Jin Long Ma, Wai Yee Chan

**Affiliations:** ^1^Center for Reproductive Medicine, Cheeloo College of Medicine, Shandong University, Jinan, China; ^2^CUHK-SDU Joint Laboratory on Reproductive Genetics, School of Biomedical Sciences, The Chinese University of Hong Kong, Hong Kong, China; ^3^National Research Center for Assisted Reproductive Technology and Reproductive Genetics, Jinan, China; ^4^SDIVF R&D Centre, Hong Kong Science and Technology Parks, Sha Tin, China; ^5^Center for Reproductive Medicine, Renji Hospital, School of Medicine, Shanghai Jiao Tong University, Shanghai, China

**Keywords:** VCD, ovotoxicity, folliculogenesis, ovulation induction, PI3K-Akt pathway

In the original article, there was a mistake in Figure 3 as published. C&E “p-rpS6(S240/242)” should be “p-rpS6(S240/244),” G “bar-headed line symbol after Bax/Bcl2” should be “arrow symbol.”

**Figure 3:**
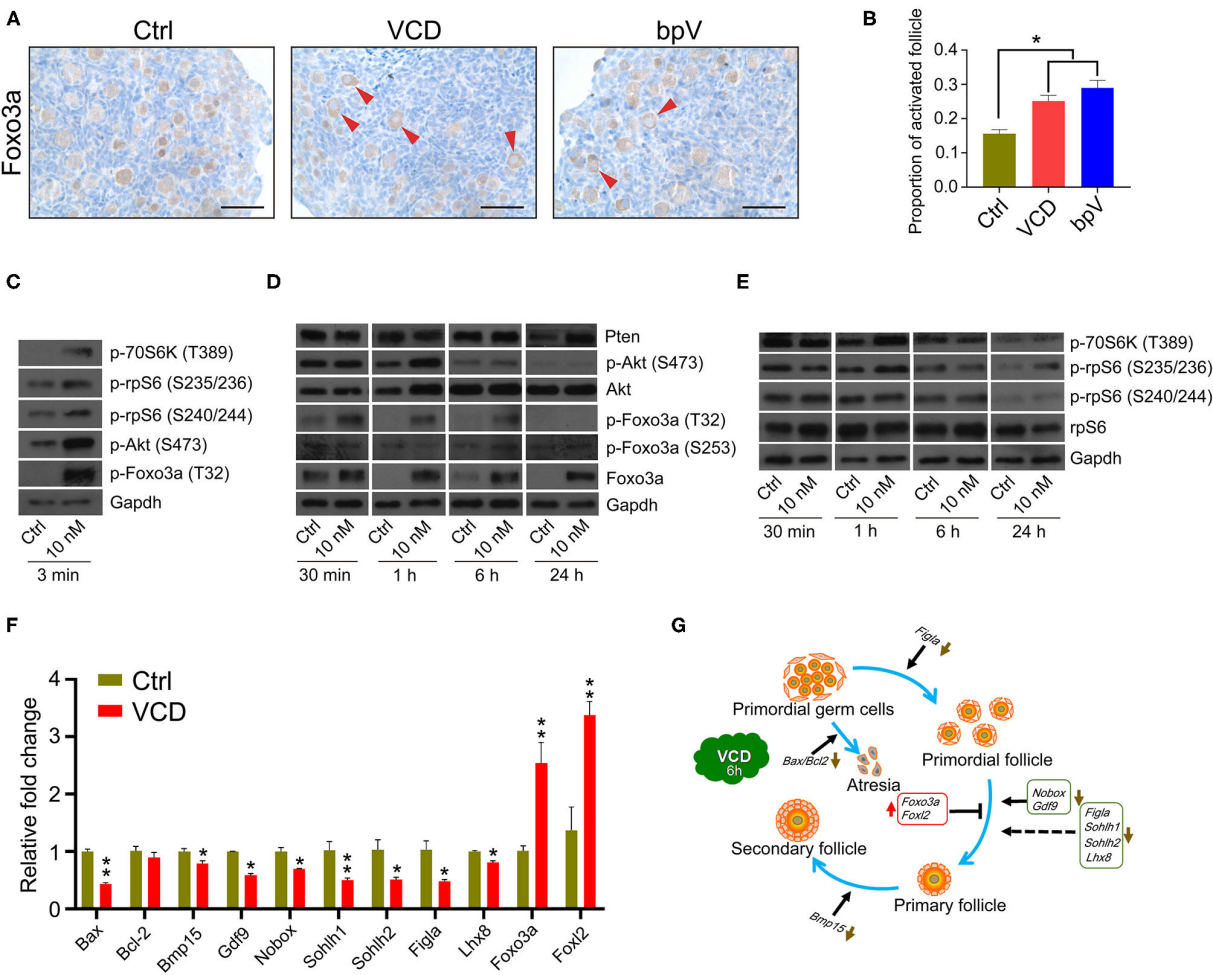
Very short-term VCD exposure transiently activates the PI3K-Akt-mTOR pathway activity, but extended exposure causes follicular development arrest. **(A)** Immunohistochemical staining of Foxo3a in cultured PD2 ovaries with or without VCD (10 nM) for 6 h. The Pten inhibitor *bpV* (100 μM) was added as a control. Scale bar = 50 μm. **(B)** Counting ratio of Foxo3a nuclear exclusion after 6 h of ovarian culture. ^*^indicates *p* < 0.05; Student's *t*-test. The data are presented as the mean ± S.D. of *n* = 6 biological replicates per group, and four tissue sections were counted per ovary. **(C)** Immunoblotting of phosphorylation levels for several proteins (P70S6k at Thr389, rpS6 at Ser235/236, rpS6 at Ser240/244, Foxo3a at Thr32, and Akt at Ser473) in cultured PD2 ovaries immediately after VCD exposure (3 min). Gapdh was used as the loading control. Representative blot images are shown, *n* = 3 biological replicates per group. **(D)** Immunoblotting of proteins of the PI3K-Akt-Foxo3a pathway (Foxo3a at Thr32, Foxo3a at Ser253, Foxo3a, Akt at Ser473, Akt, and Pten) in cultured PD2 ovaries exposed to 10 nM VCD for different times. Gapdh was used as the loading control. Representative blot images are shown, *n* = 3 biological replicates per group. **(E)** Immunoblotting of proteins of the mTOR pathway (P70S6k at Thr389, rpS6 at Ser235/236, rpS6 at Ser240/244, and rpS6) in cultured PD2 ovaries exposed to 10 nM VCD for different times. Gapdh was used as the loading control. Representative blot images are shown, *n* = 3 biological replicates per group. **(F)** qPCR expression analysis of genes related to early follicle development in cultured PD2 ovary samples treated with VCD (10 nM) for 6 h. ^*^indicates *p* < 0.05 and ^**^indicates *p* < 0.01 by Student's *t*-test, *n* = 3 biological replicates per group. **(G)** Diagram showing changes in the levels of selected transcription factors related to early follicle development after 6 h of VCD exposure.

Additionally, there was a mistake in the legend for **Figure 6** as published. H “PD22 mice” should be “PD28 mice”. The corrected legend appears below.

**Figure 6 ∣** The aromatase-promoting effects of VCD. **(A)** Immunoblotting with antibodies against aromatase and β-actin of extracts from primary ovarian GCs of PD20 mice treated with VCD (1 nM, 10 nM, 100 nM, or 1 μM) or FSH (50 ng/ml) for 24 h. **(B)** Quantitation of **(A)** using the gray value detection module of ImageJ; ^**^ indicates *p* < 0.01 compared to untreated controls by Student's *t*-test. The data are presented as the mean ± S.D. of *n* = 3 biological replicates per group. **(C,D)** The β-actin-normalized protein expression levels of aromatase in cultured PD12 ovaries exposed to 10 nM VCD for 24 h were analyzed using immunoblotting, with quantification using the ImageJ software. Representative blot images are shown. ^**^ indicates *p* < 0.01 compared to the drug-paired controls by Student's *t*-test. The data are presented as mean ± S.D. of *n* = 3 biological replicates per group. **(E,F)** The human granulosa-like tumor KGN cell line cultured in FBS-free DMEM/F12 medium exposed to VCD at 10 nM, 100 nM, 1 μM, and 10 μM or FSH (50 ng/ml) for 24 h was analyzed using immunoblotting, with quantification using the ImageJ software. Representative blot images are shown. ^**^ indicates *p* < 0.01 compared to the drug-paired controls by Student's *t*-test. The data are presented as the mean ± S.D. of *n* = 3 biological replicates per group. **(G)** Histological examination of ovarian aromatase-stained PD12 mice exposed to VCD at 10 nM or 10 μM for 24 h *in vitro*. Scale bar = 100 μm. **(H)** Histological examination of ovarian aromatase-stained PD28 mice IP injected with VCD (80 mg/kg) once each day for 5 days. Scale bar = 100 μm. **(I)** Exposure to VCD at 10 pM, 100 pM, 1 nM, 10 nM, 100 nM, 1 μM, 10 μM, or 100 μM (or PBS control) for 24 h. The estrogen levels in the KGN cell culture supernatants were measured at 24 h using ELISA. The data are presented as the mean ± S.D. of *n* = 3 biological replicates per group. **(J)** VCD exposure for 24 h (with or without the aromatase substrate testosterone at 10 nM), followed by ELISA-based measurement of estrogen levels at 24 h in the PD12 ovarian culture supernatants. PBS was the negative control and FSH (50 ng/ml) was the positive control, letrozole (Let) is used as aromatase inhibitor. The data are presented as the mean ± S.D. of *n* = 3 biological replicates per group.

In the section of “MATERIALS AND METHODS,” ^**^Paragraph 1^**^ of subsection titled ^**^Ovary Transplantation Under the Kidney Capsule^**^, “The paired ovaries (VCD-treated and paired controls) from the same donor were excised and separately allografted into two 2-month-old host mice followed by IP injection of VCD at 80 mg/ml for three consecutive days.” Should be “The paired ovaries (VCD-treated and paired controls) from the same donor were excised and separately allografted into two 2-month old host mice followed by IP injection of VCD at 80 mg/kg for three consecutive days.”

In the section of “RESULTS,” ^**^Paragraph 2^**^ of subsection titled ^**^Very Short-Term VCD Exposure Promotes the Activation of Primordial Follicles by Transiently Activating the PI3K/Akt/mTOR Pathway, Whereas Extended Exposure Prevents Further Follicular Activation^**^, “Specifically, allograft-paired ovaries from a single PD2 donor mouse were separately transplanted into two 2-month-old ovariectomized host mice, one of which was exposed to VCD (via IP injection, 80 mg/ml daily for 3 days).” Should be “Specifically, allograft-paired ovaries from a single PD2 donor mouse were separately transplanted into two 2-month-old ovariectomized host mice, one of which was exposed to VCD (via IP injection, 80 mg/kg daily for 3 days).

A correction has been made to ^**^Ovary Transplantation Under the Kidney Capsule^**^, ^**^Paragraph 1^**^ and ^**^Very Short-Term VCD Exposure Promotes the Activation of Primordial Follicles by Transiently Activating the PI3K/Akt/mTOR Pathway, Whereas Extended Exposure Prevents Further Follicular Activation^**^,^**^Paragraph 2^**^

Corrected paragraph:

“The kidneys of the anesthetized host animals were externalized through a dorso- longitudinal incision. The paired ovaries (VCD-treated and paired controls) from the same donor were excised and separately allografted into two 2-monthold host mice followed by IP injection of VCD at 80 mg/kg for three consecutive days. The age-matched paired control groups were treated with an equal amount of 0.9% normal saline. Starting at 3 days after ovary transplantation donated by PD12 mice, the hosts were administrated 2 IU of follicle-stimulating hormone (FSH) per mouse via daily IP injection, and the grafts were harvested 7 or 14 days later for histological examination. No FSH treatment was given to the host mice that received ovarian grafts from PD2 donors.”

“Pursuing the supposition that VCD might protect primordial and primary follicles from atretic degeneration, we next conducted experiments following Li et al. (2010) based on ovarian grafts from PD2 mice onto the kidney of a 2-month-old mouse. Specifically, allograft-paired ovaries from a single PD2 donor mouse were separately transplanted into two 2-month-old ovariectomized host mice, one of which was exposed to VCD (via IP injection, 80 mg/kg daily for 3 days). Upon examination at post ovarian graft day 7, the transplanted ovaries of the VCD-exposed mice appeared larger and had significantly greater numbers of secondary follicles than the transplanted ovaries of the untreated control host mice (the mean number of secondary follicles was more than 5.2-fold higher in VCD-exposed mice; *P* < 0.05). No differences were observed in the numbers of primordial or primary follicles (Figures 2C–F).

The authors apologize for this error and state that this does not change the scientific conclusions of the article in any way. The original article has been updated.
