# Supplementary figures and images for: Hormone-Like Effects of 4-Vinylcyclohexene Diepoxide on Follicular Development
Source: Front Cell Dev Biol. 2020 Jul 31;8:587. doi: 10.3389/fcell.2020.00587 (PMC7412635; doi:10.3389/fcell.2020.00587)

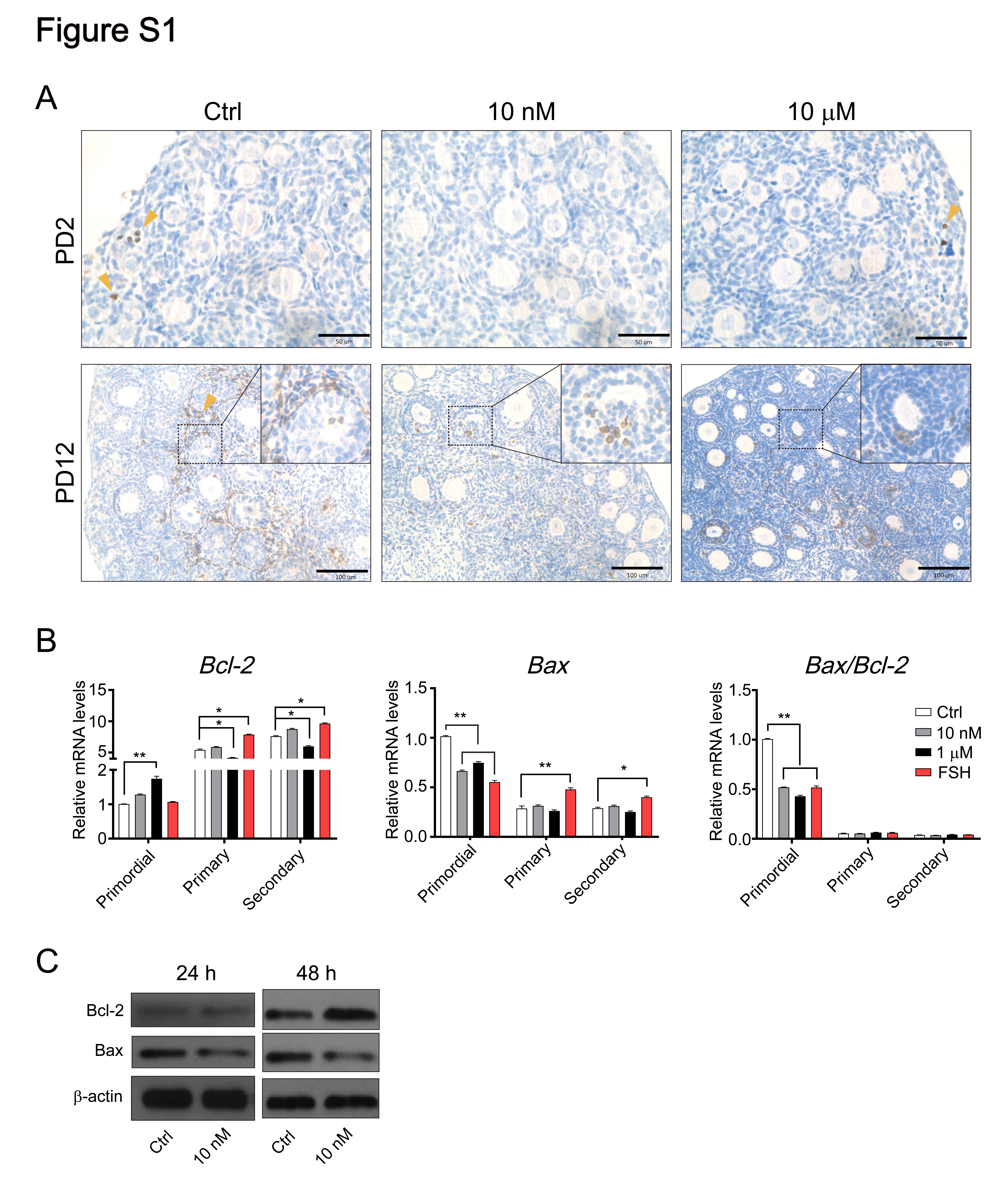

Supplement: FIGURE S1 — VCD short-term treatment reduces follicular apoptosis. (A) Immunohistochemical staining of cleaved caspase3 in cultured PD2 ovaries with or without VCD (10 nM, 10 μM) for 24 h. Yellow arrows indicate cleaved caspase-3-positive cells. PD2 ovary scale bar = 50 μm, PD12 ovary scale bar = 100 μm. (B) Quantification by staged single-oocyte qPCR of the mRNA expression of Bcl-2 and Bax in isolated primordial (20 μm), primary (40–50 μm), and secondary follicles (70 μm) derived from PD12 ovaries cultured with VCD (10 nM, 1 μM) and FSH (50 ng/ml) for 48 h. *indicates p < 0.05 and **indicates p < 0.01 by Student’s t-test, n = 3 biological replicates per group. (C) The β-actin-normalized protein expression levels of Bax and Bcl-2 in cultured PD2 ovaries exposed to 10 nM VCD for 2 days were analyzed using immunoblotting. [file Image_1.JPEG]

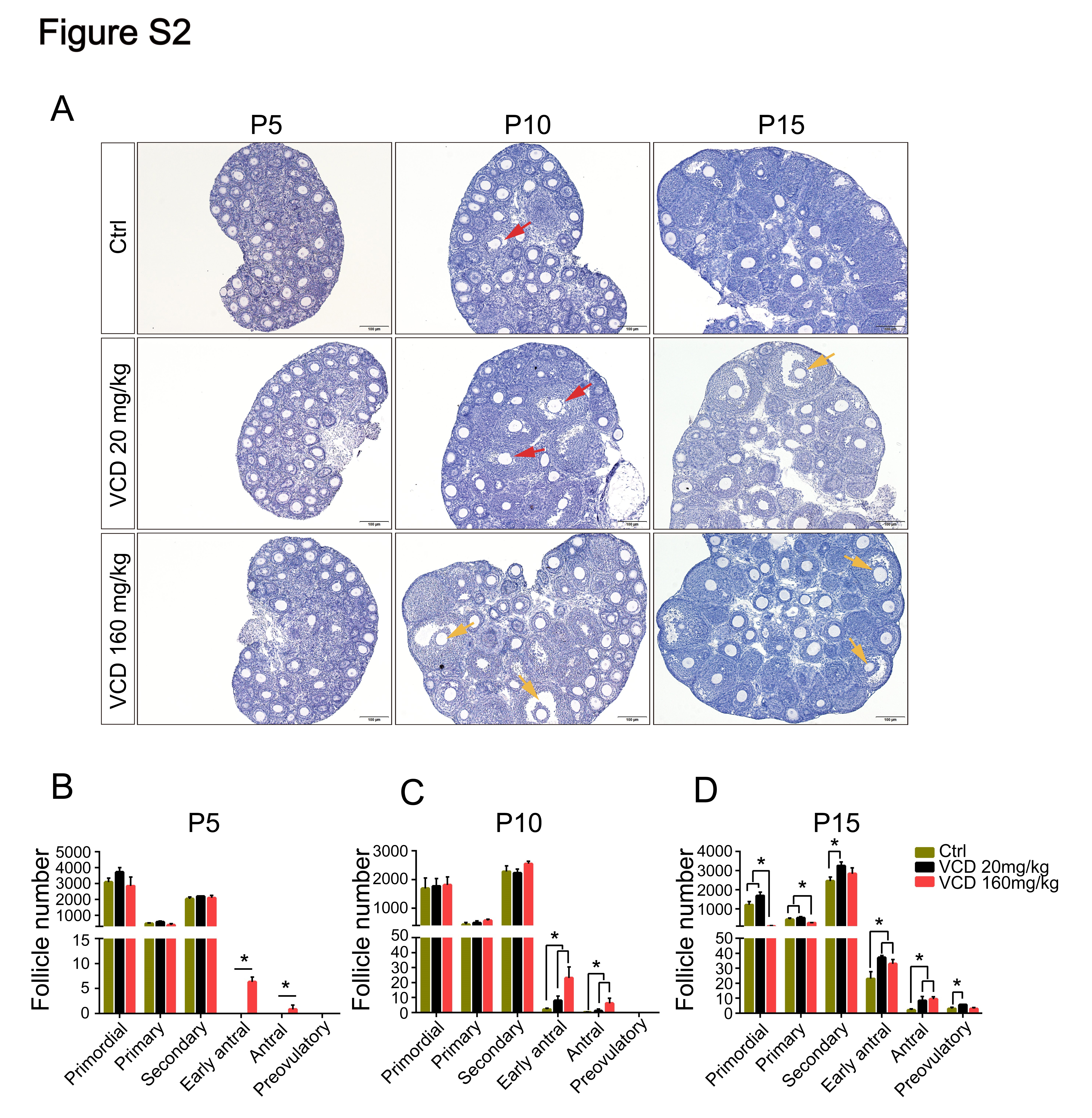

Supplement: FIGURE S2 — VCD broadly promotes follicular development in vivo. (A) Histological examination of H&E-stained ovaries from PD12 mice given IP injection of VCD (20 mg/kg or 160 mg/kg) daily for 5, 10, or 15 days. Preantral and antral follicles are marked by red and yellow arrowheads, respectively. Scale bar = 100 μm. (B–D) Quantification of staged follicles in VCD-exposed PD12 ovaries for 5, 10, and 15 days. ∗ indicates p < 0.05 compared to aged-matched controls by ANOVA. The data are presented as the mean ± S.D. of n = 3–6 biological replicates per group. [file Image_2.JPEG]

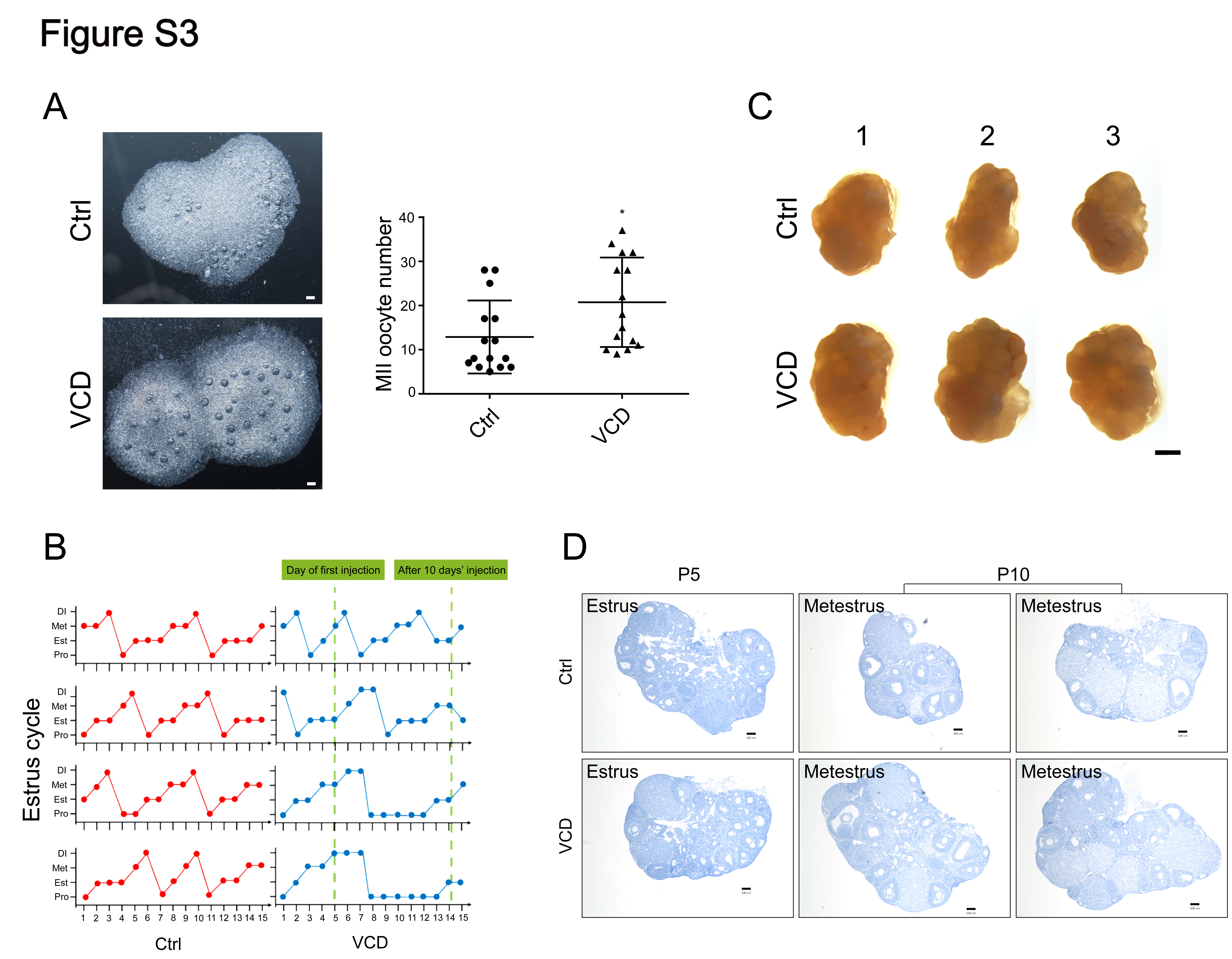

Supplement: FIGURE S3 — Impacts of VCD exposure on follicular development and ovulation in young mice. (A) After daily IP injections of VCD (80 mg/kg) for 5 days, 2-month-old young adult mice were injected IP with 5 IU of PMSG for 48 h before injection of 10 IU of human chorionic gonadotropin (hCG). Sixteen hours after the hCG injection, cumulus-oocyte complexes were collected from the fallopian tubes. Scale bar = 500 μm. Quantification of MII-stage oocytes in VCD-exposed 2-month-old mouse ovaries. *indicates p < 0.05 by Student’s t-test. The data are presented as the mean ± S.D. of n = 15 biological replicates per group. (B) With or without 10 days of IP injection of VCD (160 mg/kg) in mice, the estrous cycle at different time points was detected by vaginal lavage. (C) Two-month-old mice with or without 10 days of IP injection of VCD (80 mg/kg). Scale bar = 500 μm. (D) Histological examination of H&E-stained ovaries from 2-month-old mice exposed to VCD (80 mg/kg) via IP for 5 or 10 days. The mice were sacrificed after the final injection. Scale bar = 100 μm. [file Image_3.JPEG]

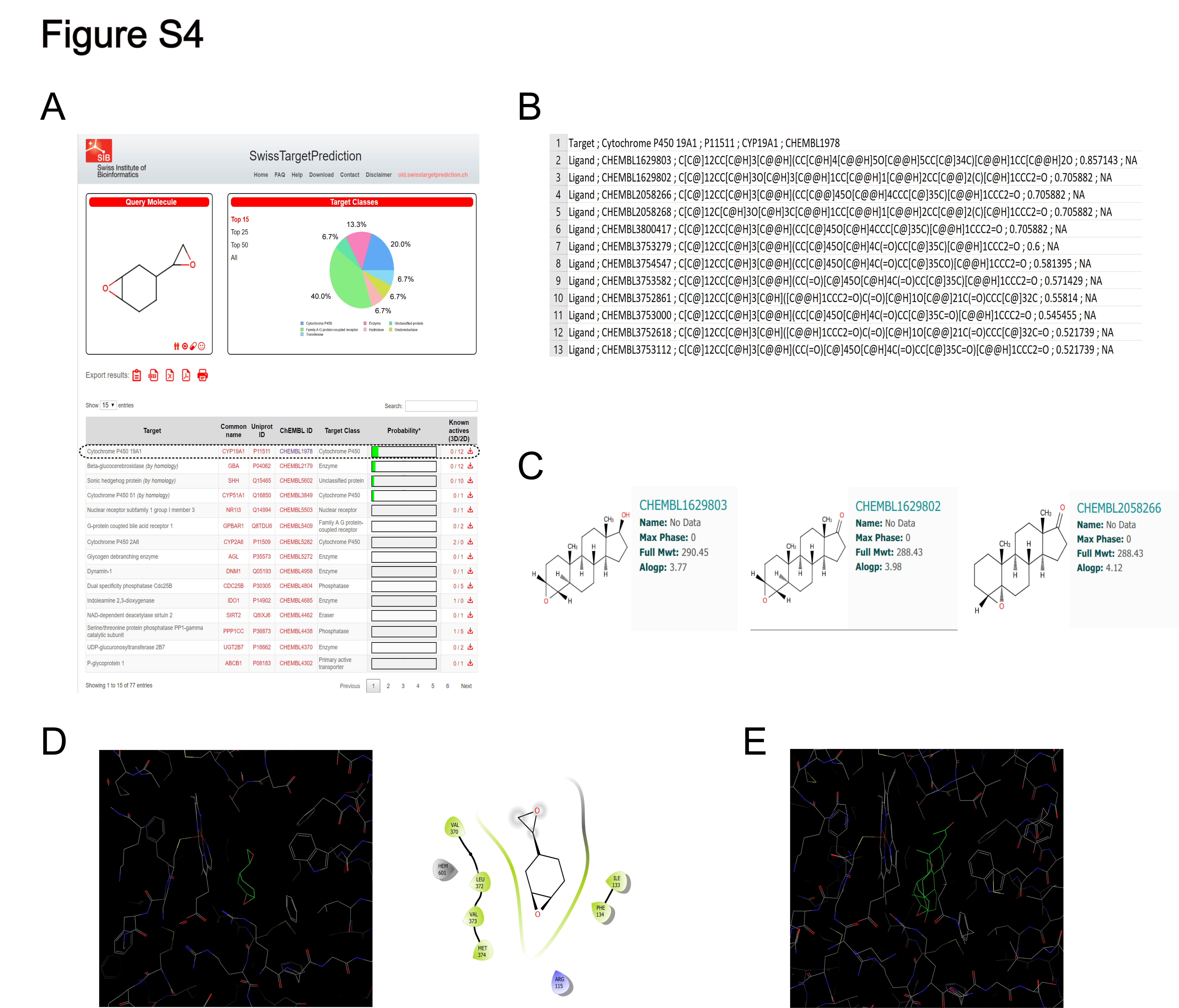

Supplement: FIGURE S4 — Computational predictions of VCD-aromatase binding. (A) Cytochrome P450 19A1 is predicted to be the primary target of VCD. (B) Twelve known small-molecule binders of Cytochrome P450 19A1, as well as a comparison of the structural similarity of these 12 compounds to VCD based on ChEMBL ID, SMILES, and 2D information. (C) Molecular structures of the top three known binders of Cytochrome P450 19A1. (D) 3D visualization of the putative binding conformation of VCD with aromatase (left), and 2D visualization of the putative binding interactions of VCD with aromatase (right). (E) 3D visualization of the putative binding conformation of VCD overlaid with the crystal binding conformation of the known substrate androstenedione. [file Image_4.JPEG]
